# The methodological quality of systematic reviews regarding the Core Outcome Set (COS) development

**DOI:** 10.1186/s12874-024-02182-w

**Published:** 2024-03-11

**Authors:** Hong Cao, Yan Chen, Zhihao Yang, Junjie Lan, Joey Sum-wing Kwong, Rui Zhang, Huaye Zhao, Linfang Hu, Jiaxue Wang, Shuimei Sun, Songsong Tan, Jinyong Cao, Rui He, Wenyi Zheng, Jiaxing Zhang

**Affiliations:** 1 School of Pharmaceutical Sciences, Guizhou University, 2708 South of Huaxi Avenue Road, Guiyang, China; 2 Department of Pharmacy, Guizhou Provincial People’s Hospital, No.83 Zhongshandong Road, Guiyang, Guizhou Province China; 3 Department of Health Services Management, Guizhou Medical University, Shansi Building, Huaxi College Town, Guiyang, China; 4 Global Health Nursing, Graduate School of Nursing Science, St. Luke’s International University, 10-1 Akashi-choChuo-Ku 104-0044, Tokyo, Japan; 5 Office of Health Insurance Administration, Guizhou Provincial People’s Hospital, No.83 Zhongshandong Road, Guiyang, Guizhou Province China; 6 Department of endoscopy, Guizhou Provincial People’s Hospital, No.83 Zhongshandong Road, Guiyang, Guizhou Province China; 7 Experimental Cancer Medicine, Department of Laboratory Medicine, Karolinska Institute, Room 601, Novum PI 6, Hälsovägen 7, SE-14157 Huddinge, Stockholm, Sweden

**Keywords:** Systematic reviews (SRs), Core outcome set (COS), Methodological research, AMSATR 2.0, Core outcome measures in effectiveness trials (COMET)

## Abstract

**Background:**

The Core Outcome Measures in Effectiveness Trials (COMET) working group proposed core outcome sets (COS) to address the heterogeneity in outcome measures in clinical studies. According to the recommendations of COMET, performing systematic reviews (SRs) usually was the first step for COS development. However, the SRs that serve as a basis for COS are not specifically appraised by organizations such as COMET regarding their quality. Here, we investigated the status of SRs related to development of COS and evaluated their methodological quality.

**Methods:**

We conducted a search on PubMed to identify SRs related to COS development published from inception to May 2022. We qualitatively summarized the disease included in SR topics, and the studies included in the SRs. We evaluated the methodological quality of the SRs using AMSTAR 2.0 and compared the overall quality of SRs with and without protocols using the Mann-Whitney U test.

**Results:**

We included 175 SRs from 23 different countries or regions, and they mainly focused on five diseases: musculoskeletal system or connective tissue disease (n = 19, 10.86%), injury, poisoning, or certain other consequences of external causes (n = 18, 10.29%), digestive system disease (n = 16, 9.14%), nervous system disease (n = 15, 8.57%), and genitourinary system disease (n = 15, 8.57%). Although 88.00% of SRs included randomized controlled trials (RCTs), only a few SRs (23.38%) employed appropriate tools to assess the risk of bias in RCTs. The assessment results on the basis of AMSTAR 2.0 indicated that most SRs (93.71%) were rated as ‘’critically low’’ to ‘’low’’ in terms of overall confidence. The overall confidence of SRs with protocols was significantly higher than that without protocols (*P* <.001). Compared to the SRs with protocols on Core Outcome Measures in Effectiveness Trials (COMET), SRs with protocols on PROSPERO were of better overall confidence (*P* = .017).

**Conclusion:**

The overall quality of published SRs regarding COS development was poor. Our findings emphasize the need for researchers to carefully select the disease topic and strictly adhere to the requirements of optimal methodology when conducting a SR for the establishment of a COS.

**Supplementary Information:**

The online version contains supplementary material available at 10.1186/s12874-024-02182-w.

## Background

Heterogeneity in outcome measures in clinical studies is widely recognized as an obstacle to evidence synthesis [1–3]. In a previous study [4], a survey was performed on 82 randomized controlled trial (RCT) protocols investigating treatment modalities for COVID-19. The study concluded that the significant heterogeneity of primary outcomes and lack of critical outcomes across these COVID-19 studies may lead to a waste of research resources. To address this issue, Jin et al. [5] developed a Core Outcome Set (COS) for different subtypes of laboratory-confirmed COVID-19 cases on the basis of two rounds of Delphi survey and one consensus meeting.

A COS refers to a minimum set of indicators that must be reported in clinical studies in specific health fields. It includes industry-recognized clinical outcomes, outcome indicators, their measurement methods and measurement time points [6]. The establishment and execution of COS can improve the value of clinical research, help researchers to identify reporting bias [1, 7, 8], and reduce the waste of research resource [9], thus facilitating evidence curation and clinical decision-making [10]. The Core Outcome Measures in Effectiveness Trials (COMET) working group which aims to develop, apply, disseminate, and update COS was founded in 2010 by internationally recognized professionals in evidence-based medicine. The COMET organization has developed a free, open-access, searchable platform for knowledge sharing and scholarly exchange. It offers methodological guidance and reporting standards for COS studies. The methods advocated by the COMET handbook [11] for COS production include systematic reviews (SRs), eDelphi, consensus meetings, semi-structured interviews, focus groups, nominal group method, among others.

SR, the best evidence for medical decision-making, is recommended by COMET as the initial step for COS development due to its capabilities of comprehensive search of literature, rigorous evaluation of evidence, and efficient curation of outcomes. The quality of SR has a direct impact on the final COS. Rogozińska et al. [12] evaluated the methods of 93 SRs (90 full reports and 3 summaries) published in the COMET database using A Measurement Tool to Assess Systematic Reviews (AMSTAR) 2.0 (items 1–9), the results of which suggest that future studies should ensure that the methods used to generate the different outcomes and outcome domains are reported transparently. Nevertheless, this study did not provide a “reliability” rating of SRs in accordance with AMSTAR 2.0. Recently, there has been an increase in the number of SRs regarding COS published on PubMed. Therefore, we conducted this overview to investigate the research status of SRs that serve as a basis for COS development and assess the quality of these SRs using complete AMSTAR 2.0.

## Methods

### Eligibility criteria

We included any study if it was a SR that constructed an outcome pool for the COS development. We excluded SR protocols, SRs without full text or detailed information, and SRs published in languages other than English or Chinese.

### Literature search

We conducted a search on PubMed from its earliest records to May 30, 2022 to identify published SRs related to the development of COS. We used Medical Subject Headings and free text terms associated with “core outcome set” or “COS” to search for relevant studies.

### Study selection and data extraction

Two investigators (H. C and Y. C), who were trained in research methods and had experiences in SRs related to COS, independently screened the titles and abstracts of the SRs for inclusion and subsequently reviewed the full texts of the selected studies against pre-defined inclusion and exclusion criteria. They also independently extracted the following data from included SRs: (1) general information, such as the first author, year of publication, country, registry number, disease population, interventions, outcomes, and their definitions; and (2) methodological information, such as database searched, tools used for assessing the quality of evidence, types of included studies, the method used to develop the COS, and so on. Any disagreement in the process of study selection and data extraction was resolved by discussion.

### Quality assessment

Two investigators (H. C and Y. C) appraised the methodological quality of each included SR independently using the AMSTAR 2.0 [13], and any discrepancies were resolved through discussion. AMSTAR 2.0 comprises 16 items, with Item 2, 4, 7, 9, 11, 13, and 15 being critical items that significantly impact the review’s validity and conclusion. The project’s assessment is either “Yes” or “no” for Item 1, 3, 5, 6, 10, 13, 14, and 16; “Yes”, “Partial Yes” or “No” for Item 2, 4, 7, 8, and 9; or “Yes”, “No”, or “No meta-analysis” for Item 11, 12, and 15. There are four levels of quality for a SR: “high”, “moderate”, “low”, and “critically low” in terms of “no or one non-critical weakness”, “more than one non-critical weakness”, “one critical flaw with or without non-critical weakness”, and “more than one critical flaw with or without non-critical weakness”, respectively.

### Statistical analysis

We provided a qualitative summary of the disease categories covered in the SR topics, the study design types included in the SRs, pathways for COS development, and the methodological quality of the SRs. Categorical variables were presented as frequency and percentage, while continuous variables were described using mean (and standard deviation) or median (and interquartile range (IQR)). We used the methodological quality of the SRs as an ordinal variable, categorized as “high”, “moderate”, “low”, or “critically low”. We employed the Mann-Whitney U test to compare the methodological quality of SRs with and without protocols and to evaluate the impact of different registered protocols (PROSPERO [14] vs. COMET) on the methodological quality of SRs. R software (version 3.6.3) was applied for data analysis. *P* ≤ .05 was considered statistically significant, and all tests were two-sided.

To assess the agreement between the two reviewers in study selection and quality assessment, we calculated the kappa statistic (*K*). The value of *K* ranges from 0 to 1, where values of 0 to 0.20, 0.21 to 0.39, 0.40 to 0.59, 0.60 to 0.79, 0.80 to 0.90, and above 0.90 represent no agreement, minimal agreement, weak agreement, moderate agreement, strong agreement, and almost perfect agreement, respectively [15].

## Results

### Search results and description of included SRs

**Fig. 1:**
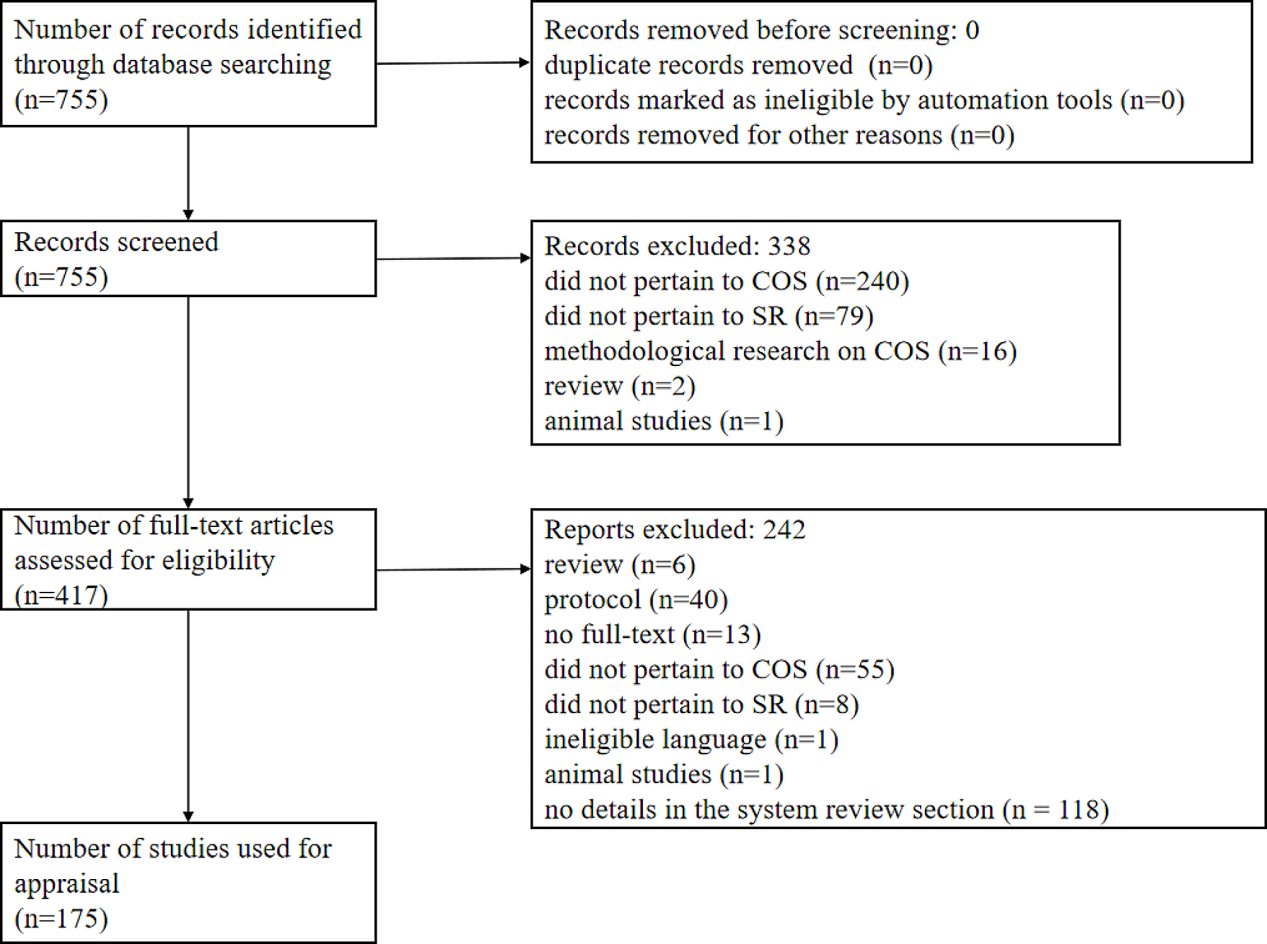
Flow diagram of study selection process for this study. SR: systematic review; COS: core outcome set

A total of 755 studies were initially retrieved from PubMed. Following the selection process (see Figs. 1), 175 SRs were included in the final assessment. The level of agreement between the two reviewers for study selection was acceptable (*K* = 0.89). As presented in Table S1, the included SRs were published between 2006 and 2022, with a marked increase in quantity after 2018. The authors of the SRs were from 23 countries or regions, with 42.86% of authors hailing from the United Kingdom.

**Fig. 2:**
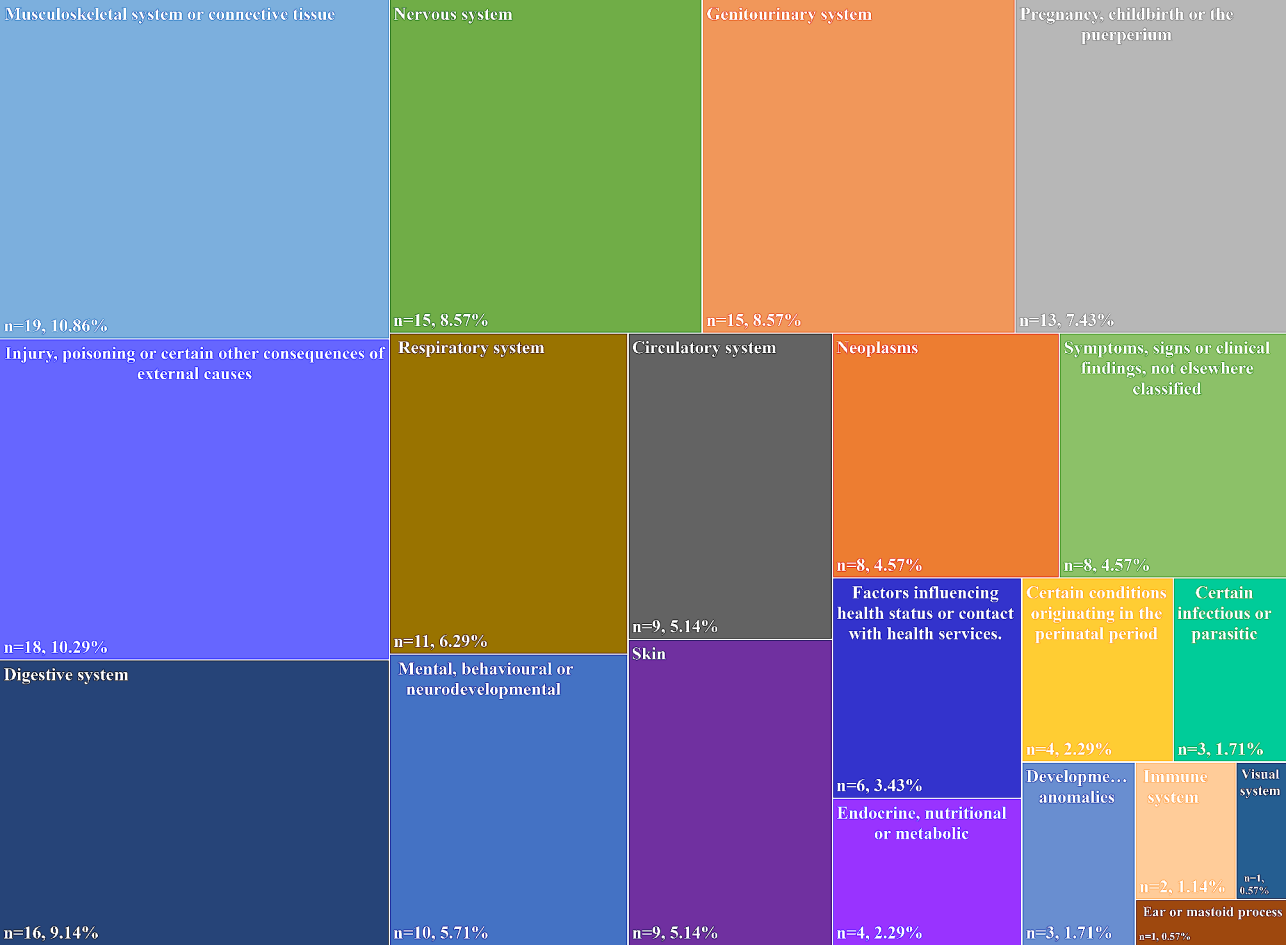
Disease distribution for constructing core outcome set

Based on the International Classification of Diseases (ICD)-11 [16] (Fig. 2), the topics of the 175 included SRs were focused on 20 different types of diseases. The top five diseases with the highest number of literature were musculoskeletal system or connective tissue disease (*n* = 19, 10.86%), injury, poisoning, or certain other consequences of external causes (*n* = 18, 10.29%), digestive system disease (*n* = 16, 9.14%), nervous system disease (*n* = 15, 8.57%), and genitourinary system disease (*n* = 15, 8.57%).

**Fig. 3:**
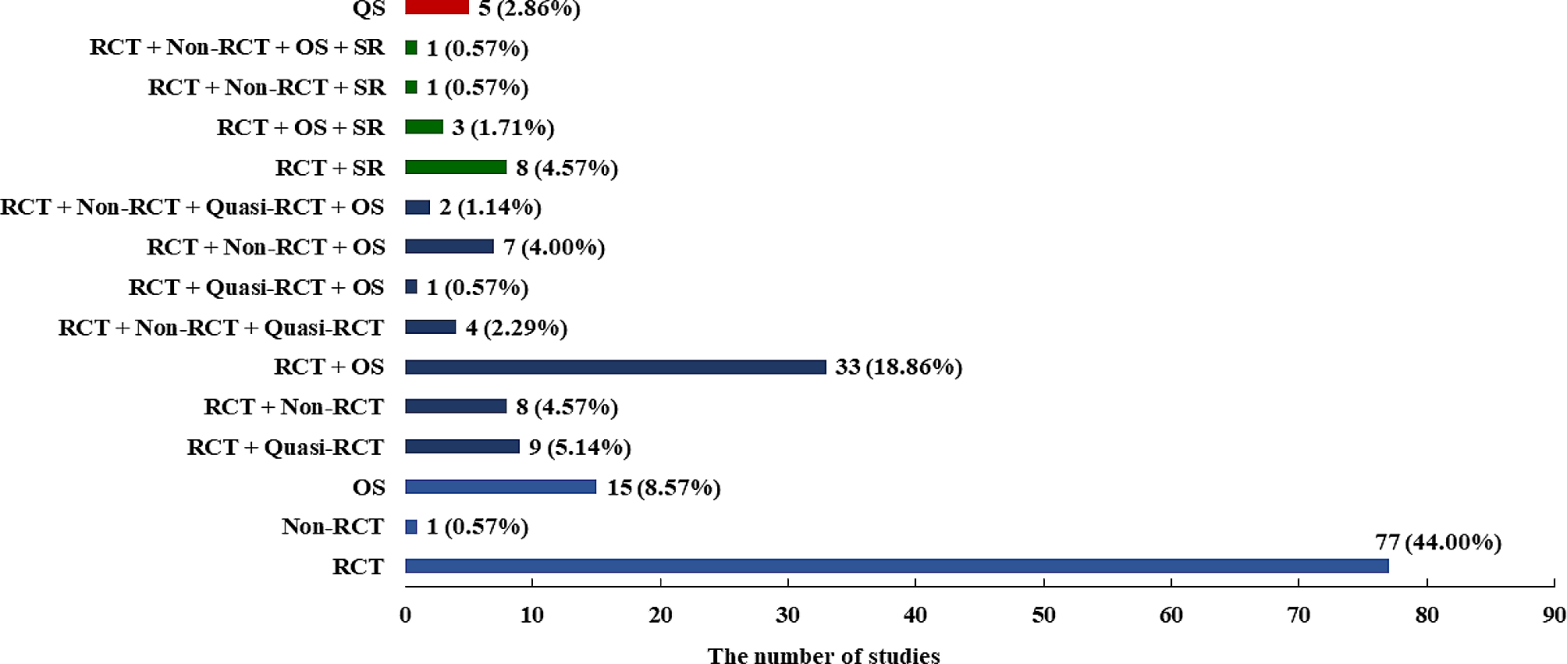
Design types of studies included in the systematic reviews. QS: Qualitative methods; SR: systematic review; RCT: randomized controlled trial; Quasi-RCT: quasi-randomized controlled trial; Non-RCT: non-randomized controlled trial; OS: Observational study

Figure 3 shows that out of the 175 SRs included in the final assessment, 98 SRs (56.00%) included only one type of study. Among these, randomized controlled trials (RCTs) were the most common type (*n* = 77, 44.00%), followed by observational studies (*n* = 15, 8.57%), non-randomized controlled trials (*n* = 1, 0.57%), and qualitative studies (*n* = 5, 2.86%). Additionally, 162 SRs (92.57%) only included primary research, such as RCTs, quasi-randomized controlled trials, non-RCTs, cohort studies, case-control studies, cross-sectional studies, and qualitative research. Thirteen SRs (7.43%) included both primary research and SR. While 154 SRs (88.00%) selected RCTs as the included studies, only 52 out of 154 (33.12%) SRs assessed the methodological quality of the included RCTs. Among these, only 36 out of 154 (23.38%) SRs used appropriate tools to assess the risk of bias in RCTs.

**Fig. 4:**
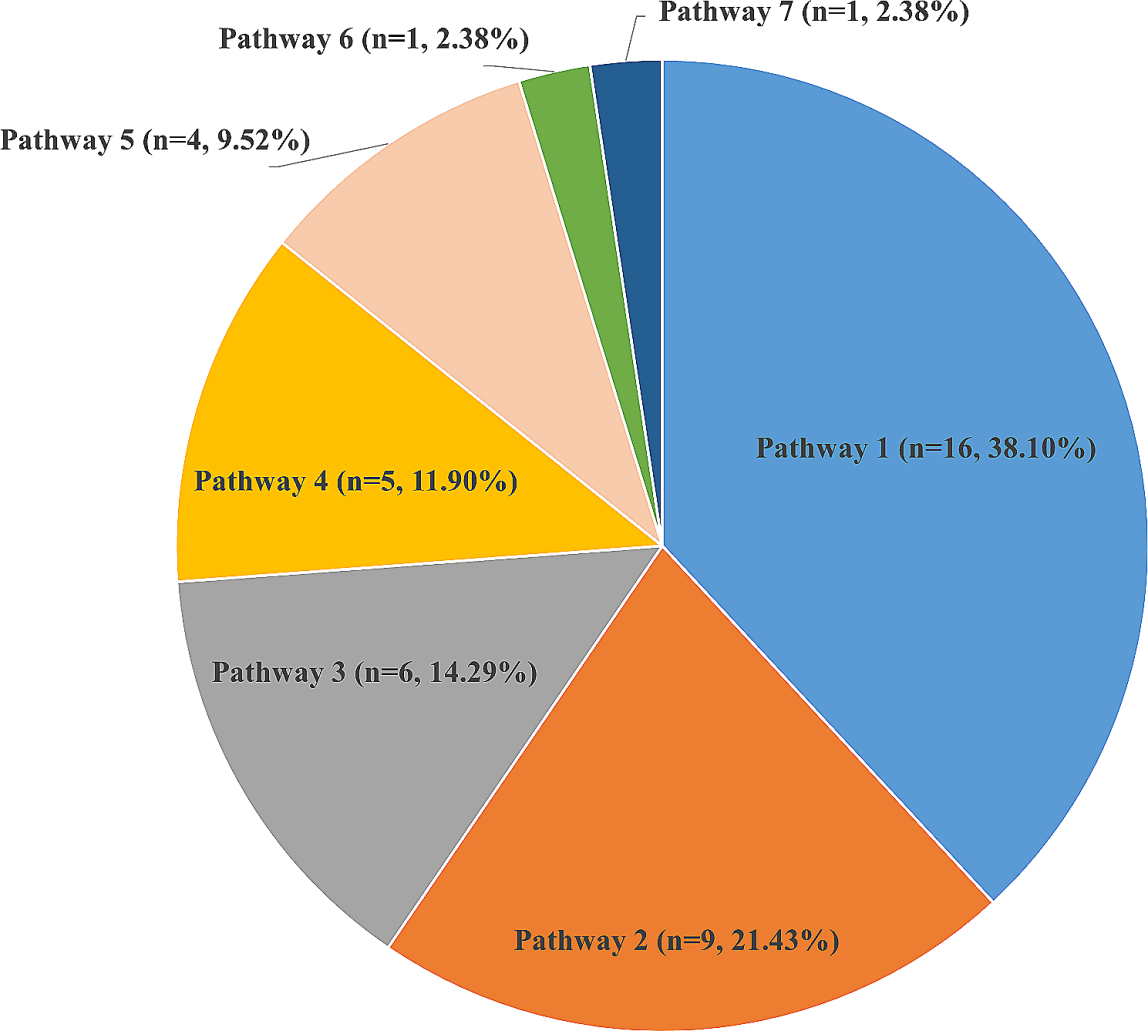
Methodological pathway for developing core outcome set. Pathway 1: “(1)systematic review, (2)semi-structured interview, (3)Delphi survey, (4)consensus meeting”; Pathway 2: “(1)systematic review, (2)Delphi survey”; Pathway 3: “(1)systematic review, (2)Delphi survey, (3)consensus meeting”; Pathway 4: “(1)systematic review, (2) focus group, (3)Delphi survey, (4)consensus meeting”; Pathway 5: “(1)systematic review, (2)semi-structured interview, (3)focus group, (4)Delphi survey, (5)consensus meeting”; Pathway 6: “(1)systematic review, (2)semi-structured interview, (3)focus group, (4)Delphi survey/consensus meeting”; Pathway 7: “(1)systematic review, (2)focus group, (3) consensus meeting”

A total of 42 SRs reported 7 different methodological pathways for COS development (Table S2 and Fig. 4). Among these, most studies (16 out of 42, 38.10%) selected a research approach consisting of a systematic review, semi-structured interview, Delphi survey, and consensus meeting for COS development. Delphi survey and consensus meeting were the most common methods used for COS development, in addition to systematic reviews.

### Methodological quality of included SRs

**Fig. 5:**
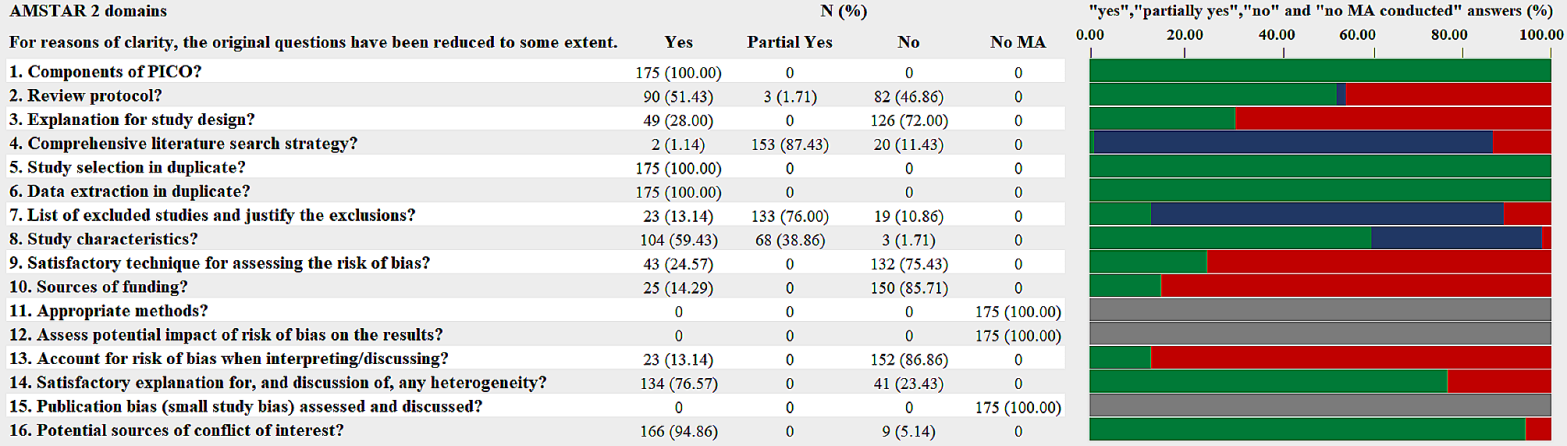
Methodological quality of included systematic reviews according to the AMSTAR 2.0. Green: “yes”; Blue: “partially yes”; Red: “no”; Grey: “not applicable due to no meta-analysis conducted”

The results of methodological quality assessment of included SRs were shown in accordance with AMSTAR 2.0 [13] (see Fig. 5). The level of agreement between the two reviewers for quality assessment was acceptable (*K* = 0.81 ~ 0.93 for all items and overall confidence). Since none of the SRs performed meta-analysis, Item 11, Item 12, and Item 15 were not applicable in the final assessment. All SRs provided the Population, Intervention, Comparator group, and Outcome (PICO) and performed study selection and data extraction in duplicate, hence they were rated as “yes” in Item 1, Item 5, and Item 6, respectively. Regarding Item 16, most SRs (166 out of 175, 94.86%) were rated “yes” since there were no competing interests in these studies.

Despite 93 SRs (53.14%) having written protocols, only 90 SRs were rated “yes” in Item 2 because 3 of them did not register or publish the protocols before the studies were initiated. Regarding Item 3, only 49 SRs (28.00%) explained the reason for including RCTs or other observational studies. 155 SRs searched at least 2 databases, and the search strategy was provided along with the justification for publication restrictions. However, out of the 155 SRs, only 2 SRs searched the reference lists or bibliographies of included studies, trial or study registries, and grey literature. As a result, only these 2 SRs were rated “yes” for Item 4 on AMSTAR 2.0, while the remaining 153 were rated “partially yes”.

In terms of Item 7, 133 SRs (76.00%) provided a list of excluded studies and the main reasons and were rated “partial yes”, while 23 SRs (13.14%) explained the exclusion reason for each study and were rated “yes”. Nearly all SRs (172 out of 175, 98.28%) described the PICO and research designs of included studies, but only 104 of them provided the details about the above information and were assessed as “yes” in Item 8. Forty-three SRs (24.57%) used appropriate tools (e.g., Cochrane Risk-of-Bias tool (RoB) 1.0 for randomized trials [17], JADAD scale [18], a criteria list for quality assessment of RCTs [19], Critical Appraisal Skills Program (CASP) checklist [20], The Evidence Project risk of bias tool [21], Oxford Centre for Evidence-Based Medicine Levels of Evidence [22], etc.) to assess the risk of bias in individual studies included in the SRs, hence they were rated “yes” in Item 9.

In Item 10, 150 SRs (85.71%) did not report on the sources of funding for individual studies included in the review and were evaluated as “no”. Due to the discussion about the impact of the risk of bias on the results, 23 SRs (13.14%) were rated “yes” in Item 13. In the presence of heterogeneity, the review authors of 134 SRs (76.57%) investigated sources of any heterogeneity in the results and discussed the impact of this on the results of the review, hence these SRs were rated “yes” in Item 14.

In summary, only 6 SRs (3.43%) were evaluated as “high” in terms of overall confidence according to the AMSTAR 2.0, while most SRs (164 out of 175, 93.71%) were “critically low” to “low”. Table 1 indicates that the overall confidence of SRs with protocols was significantly higher than those without protocols (*P* < .001). Among the SRs with protocols, 67 were registered on the PROSPERO (an international database of prospectively registered systematic reviews) and 21 on the COMET database. SRs with protocols on the PROSPERO had a higher overall confidence compared to those with protocols on the COMET (*P* = .017).

**Table 1:** Overall confidence of systematic reviews with or without protocols

The overall confidence	SRs with protocols (*n* = 93)	SRs without protocol (*n* = 82)	P-value	SRs with protocols on the PROSPERO (*n* = 67)	SRs with protocols on the COMET (*n* = 21)	P-value
**High**	6 (6.45%)	0 (0.00%)	0.000 ^*^	6 (8.96%)	0 (0.00%)	0.017 ^*^
**Moderate**	5 (5.38%)	0 (0.00%)		5 (7.46%)	0 (0.00%)	
**Low**	17 (18.28%)	5 (6.10%)		13 (19.40%)	2 (9.52%)	
**Critically Low**	65 (69.89%)	77 (93.90%)		43 (64.18%)	19 (90.48%)	

SR: systematic review; ^*^: *P* ≤ .05

## Discussion

### Main findings and interpretations

In this study, we investigated 175 SRs from 23 countries or regions regarding the development of COS. We found that the research hotspots for COS in SRs were musculoskeletal system or connective tissue disease, injury, poisoning, or certain other consequences of external causes, digestive system disease, nervous system disease, and genitourinary system disease, accounting for nearly half of all the SRs. While RCTs were the most common study design included in most SRs, only a few studies (23.38%) used appropriate tools to assess their risk of bias. In terms of COS development, most studies used SRs, Delphi surveys, and consensus meetings as components of the research pathway. According to the data from COMET website, 75 out of 175 SRs have developed their COS, while the COS for 22 SRs are still under development. Consistent with AMSTAR 2.0, the overall confidence of most SRs (93.71%) was evaluated as ‘’critically low’’ to ‘’low’’. Nevertheless, registering a protocol on a specialized database such as PROSPERO before conducting an SR could help improve the overall quality of the SRs.

The Global Burden of Disease (GBD) 2019 Diseases and Injuries Collaborators [23] conducted an analysis of 369 diseases and injuries across 204 countries and territories. They reported that the top five diseases with the highest disability-adjusted life-years (DALYs) were neonatal disorders, ischemic heart disease, stroke, lower respiratory infections, and diarrheal diseases. However, these diseases were not the focus of the included SRs. Notably, no SR was found regarding the establishment of COS for ischemic heart disease and diarrheal diseases. Additionally, only a few SRs were related to the construction of COS for neonatal disorders (*n* = 3, 1.71%), stroke (*n* = 1, 0.57%), and lower respiratory infections (*n* = 2, 1.14%). Therefore, we suggest that researchers should pay more attention to these diseases with high DALYs and focus on establishing their COS by conducting SRs.

Although assessing the risk of bias in included studies is considered the most critical step in SRs, only one-fourth of the SRs that included RCTs employed appropriate tools to assess the risk of bias in RCTs. The most common tools used in the SRs were the JADAD scale and RoB 1.0 for randomized trials. The JADAD scale, a valid tool to initially assess the quality of an RCT, has the main advantage of requiring a short amount of time to complete. However, in view of its broad assessment without key information on potential confounding factors affecting the validity of findings (such as allocation concealment, industry sponsorship, and conflict of interest), the accuracy and precision of JADAD are inferior to other more exhaustive updated instruments. ROB is the most updated, reliable, valid, and comprehensive tool and considered as the gold standard for assessing potential biases in RCTs. An overview [24] comparing the advantages and disadvantages of the JADAD scale over RoB 2.0 suggested that RoB 2.0 should be primarily considered when assessing the quality of RCTs.

The assessment on the basis of AMSTAR 2.0 revealed that the overall quality of SRs in this field was poor, which could compromise the reliability of COS. Our survey identified several methodological issues, including: (1) more than 50% of SRs did not select appropriate tools to assess the risk of bias in the included studies, account for the risk of bias in the interpretation, report the funding sources of included studies, or explain the reason for the design of included studies; (2) 20–50% of SRs did not register their protocol before starting the studies or discuss the sources of any heterogeneity and their influence on the results; (3) less than 20% of SRs did not use a comprehensive literature search strategy, provide a list of excluded studies with their reasons for exclusion, describe the included studies in adequate detail, or report any potential sources of conflict of interest. Rogozińska et al. [12] previously found that none of the SRs published on the COMET database met all nine methodological expectations for SRs outlined in AMSTAR 2.0, which was similar to our findings. Therefore, we urge researchers in this field to strive to improve the quality of SRs regarding COS by addressing the above-mentioned issues.

Additionally, we discovered that SRs with registered protocols had a significantly higher overall quality than those without. Furthermore, registering protocols on PROSPERO, as opposed to COMET, can be advantageous in improving the quality of SRs. SRs are generally considered the best evidence for addressing health research questions due to their rigorous and methodical approach, which requires adherence to pre-specified methods and analyses. Creating and registering a protocol that outlines the rationale, hypothesis, and planned methods of the SRs in the initial stage of the study ensures the transparency and reproducibility, reducing the risk of selective reporting bias. Protocols guide decisions about including/excluding studies/data in SRs during the research process and reporting outcomes in the final manuscript. As a solution to improve incomplete and biased reporting of SRs, the Preferred Reporting Items for Systematic reviews and Meta-analyses (PRISMA) guideline proposed registering protocols in 2009 [25]. In 2011, PROSPERO, the first international registry for systematic reviews, was launched by the Center for Reviews and Dissemination (CRD) at York University. The establishment of PROSPERO aimed to provide a registry for SRs in health, promote their quality, and reduce redundancy and resource waste. Wanderley et al. [26] also agreed that SRs registered with PROSPERO have the highest level of consistency and credibility. Therefore, we recommend that authors of SRs on COS should register a detailed protocol on PROSPERO before beginning the review.

### Recommendations for SRs regarding the COS development

In order to establish a convincing COS, it is essential to perform a SR with adequate methodological quality. To promote the overall quality of SRs, researchers should first register a protocol on specialized databases like PROSPERO before conducting the SR. Secondly, appropriate tools for assessing the risk of bias in the included studies should be selected, with RoB being the optimum choice for SRs that include RCTs. Improving the quality of SRs also requires amelioration of the methodological process in the following aspects: building a comprehensive literature search strategy, providing a detailed exclusion list with reasons, and accounting for the risk of bias in the results. Regarding the selection of the disease topic of SRs for COS development, researchers should focus on diseases with high DALYs that have not been covered in previous SRs regarding COS, such as ischemic heart disease and diarrheal diseases.

### Strengths and limitations

This study has several strengths, including its comprehensive and systematic investigation of methodological details about SRs regarding the COS development. Furthermore, the study had a large sample size of 175 SRs published in PubMed from the earliest to 2022, making it relatively representative.

Nonetheless, there are several limitations that should be considered. First, we only searched for eligible SRs published in PubMed and included those published in Chinese and English, potentially missing SRs that were not indexed in PubMed at the time of the search or those published in other languages. Second, despite the assistance of trained assessors, some misinterpretation may still exist in some extracted items from the original article source, which could have affected the results. Finally, the assessment of methodological quality and identification of specific methodological gaps were limited by the insufficient reporting of methodological details in some of the included SRs.

## Conclusions

In summary, despite the increasing number of SRs published on COS development, their overall quality was found to be poor. Methodological issues were present in every aspect of design and execution when examining using AMSTAR 2.0. Identifying and understanding these important methodological gaps is critical for future SRs. We have identified several critical methodological issues and provided recommendations for improving the quality of SRs on COS development. Our findings emphasize the need for researchers to carefully select the disease topic and strictly adhere to optimal methodology requirements when conducting SRs to establish COS.

### Electronic supplementary material

Below is the link to the electronic supplementary material.

Supplementary Material 1

## Data Availability

All data generated or analysed during this study are included in this published article and its supplementary information files.

## Abbreviations

COMET: Core Outcome Measures in Effectiveness Trials
COS: Core Outcome Set
SRs: Systematic Reviews
AMSTAR: A Measure Tool to Assess Systematic
RCT: Randomized Controlled Trial
COVID-19: Corona Virus Disease 2019
ICD: International Classification of Diseases
PICO: Population, Intervention, Comparator group, and Outcome
RoB: Cochrane Risk-of-Bias tool
CASP: Critical Appraisal Skills Program
GBD: Global Burden of Disease
DALYs: Disability-Adjusted Life-Years
