# Supplementary material for: The methodological quality of systematic reviews regarding the Core Outcome Set (COS) development
Source: BMC Med Res Methodol. 2024 Mar 11;24:65. doi: 10.1186/s12874-024-02182-w (PMC10926669; doi:10.1186/s12874-024-02182-w)
Supplement: Supplementary file 1 — Supplementary Material 1 [file 12874_2024_2182_MOESM1_ESM.docx]

# Table S1: Characteristics of included Systematic Reviews

| **Study** | **Year** | **author’s original country** | **Protocol Number** | **Types of disease** | **Population** | **Search Database** | **Tools for Quality Assessment** | **Types of studies included** |
| --- | --- | --- | --- | --- | --- | --- | --- | --- |
| Henry 2022^[1]^ | 2022 | Ireland | COMET study ID 2028 | A | Sepsis | Medline, Embase | none | ① |
| Barnes 2019^[2]^ | 2019 | The United Kingdom | none | A | Sepsis | Medline, Embase, Cochrane | none | ①⑧ |
| Lee, S 2019^[3]^ | 2019 | South Korea | none | A | Tuberculosis | Medline, Embase, Cochrane | Critical Appraisal Skills Program (CASP) checklist | ①⑧ |
| Lee, A 2021^[4]^ | 2021 | The United Kingdom | CRD42020200365 | B | Breast carcinoma | Medline, Embase, Cinahl | none | ④⑤ |
| Potter 2010^[5]^ | 2010 | The United Kingdom | none | B | Breast cancer | Medline, Embase, AMED, PsycINFO | Cochrane Risk-of-Bias tool for randomized trials | ①④⑦ |
| Fledderus 2019^[6]^ | 2019 | The Netherlands | CRD42019126299 | B | Congenital melanocytic naevi | Embase, PubMed, Cochrane | none | ① |
| Amoah 2022^[7]^ | 2022 | The United Kingdom | CRD42020213771 | B | Uterine myoma | Embase, Medline, PubMed, Cinahl | JADAD scale | ① |
| O’Connor 2021^[8]^ | 2021 | Ireland | none | B | Oesophagogastric cancer | Embase, PubMed, CINHAL, Cochrane, SCOPUS, PEDro | none | ①② |
| Reynolds 2021^[9]^ | 2021 | The United States | none | B | Cutaneous squamous cell carcinoma | PubMed, Embase | none | ④ |
| Alkhaffaf 2018^[10]^ | 2018 | The United Kingdom | none | B | Gastric cancer | Evidence Based Medicine, Medline, Embase, Cinahl | none | ① |
| Fish 2018^[11]^ | 2018 | The United Kingdom | CRD42016036540 | B | Anal cancer | Medline, Embase, Cochrane Central, CinahlASCC | none | ①④⑥⑩ |
| Aiyegbusi 2022^[12]^ | 2022 | The United Kingdom | none | C | Immune-mediated inflammatory diseases | COMET, ICHOM, FDA, EMA | COS–Standards for Development recommendations (COS–STAD) checklist | ⑧ |
| Harman 2021^[13]^ | 2021 | The United Kingdom | COMET study ID 1952 | C | Pulmonar sarcoidosis | ClinicalTrials. gov, ICTRP | none | ① |
| Matvienko-sikar 2018^[14]^ | 2018 | Ireland | CRD42017055608 | D | Childhood obesity | Embase, Medline, Cinahl, Cochrane, PsychINFO | COSMIN checklist (six of these questions) | ①③ |
| Hopkins 2014^[15]^ | 2014 | The United Kingdom | none | D | Obesity patients | Medline, Embase, Cochrane | none | ①②④⑦ |
| Li 2021^[16]^ | 2021 | China | COMET study ID 1891 | D | Hyperlipidemia | Cochrane, PubMed, Embase, Wan fang, CNKI, CBM | none | ①④⑤⑥⑨ |
| Harman 2017^[17]^ | 2017 | The United Kingdom | COMET study ID 956 | D | Type 2 diabetes | ClinicalTrials. gov | none | ① |
| Agar 2021^[18]^ | 2021 | Australia | none | E | Delirium | Medline, Cinahl, Embase, PsycINFO, Web of Science, Cochrane, PROSPERO, Joanna Briggs | MOMENT study scoring system/Cochrane Risk-of-Bias tool for randomized trials | ①②③ |
| Rose 2021^[19]^ | 2021 | The United Kingdom | none | E | Delirium | 10 Databases | none | ①②③ |
| Rose 2020^[20]^ | 2020 | Canada | none | E | Delirium | Medline, Medline, Cinahl, Embase , PsychINFO, Cochrane, PROSPERO, Joanna Briggs | Cochrane Risk-of-Bias tool for randomized trials | ①②③ |
| Goncalves 2018^[21]^ | 2018 | The United Kingdom | COMET study ID 708 | E | Dementia | Medline, Psycinfo, Cinahl, Web of Science, Scopus, ScienceDirect | Mixed Methods Assessment tool—version 2011 | ⑪ |
| Webster 2017^[22]^ | 2017 | The United Kingdom | CRD42015027346 | E | Dementia | Medline, ALOIS, Central, Cinahl, Embase, LILACS, PsycINFO, ClinicalTrials. gov | none | ① |
| Hellberg 2021^[23]^ | 2021 | Sweden | none | E | Perinatal depression | Medline, Cochrane , PsycINFO, Cinahl, ClinicalTrials. gov | none | ①⑥ |
| Mew 2020^[24]^ | 2020 | Canada | none | E | Adolescent depression | Medline, PsycINFO, Cochrane | none | ① |
| Kelly 2020^[25]^ | 2020 | Canada | none | E | Neonatal opioid withdrawal syndrome | *NR* | none | ①④⑦⑨ |
| Shan 2020^[26]^ | 2020 | Canada | COMET study ID 1546 | E | Neonates experiencing withdrawal | Medline, Embase, Cochrane, ClinicalTrials. gov. | none | ①②④⑤⑧⑨ |
| Addington 2012^[27]^ | 2012 | Canada | none | E | Schizophrenia | Medline, PsycINFO, PubMed, Cinahl, HealthSTAR | none | ① |
| Kapadia 2016^[28]^ | 2016 | Canada | COMET study ID 736 | F | Feeding tubes and neurologic impairment | Medline, Embase, Cochrane | none | ①④⑩ |
| Morris 2014^[29]^ | 2014 | The United Kingdom | none | F | Children with neurodisability | Medline, Embase, PsycINFO | COSMIN checklist | ①⑦ |
| Cup 2007^[30]^ | 2007 | The United States | none | F | Neuromuscular diseases | Cinahl WebSPIRS 5.1, Embase WebSPIRS 5.03, Cochrane | Cochrane Risk-of-Bias tool for randomized trials | ① |
| Deckert 2015^[31]^ | 2015 | Germany | none | F | Chronic pain | Medline, Embase, AMED | none | ①② |
| Gallo 2020^[32]^ | 2020 | Canada | CRD42017067855 | F | Cubital tunnel syndrome | Medline, Embase | none | ① |
| Grieve 2015^[33]^ | 2015 | The United Kingdom | none | F | Complex regional pain syndrome | Embase, Medline, PsycInfo, Cinahl, LILACS | none | ①⑧ |
| Hatemi 2014^[34]^ | 2014 | Turkey | none | F | Behçets syndrome | PubMed | none | ①②④⑤⑧ |
| Jerosch-herold 2006^[35]^ | 2006 | The United Kingdom | none | F | Carpal tunnel syndrome | Medline, Cinahl, AMED | none | ①③ |
| Tooren 2022^[36]^ | 2022 | The United Kingdom | none | F | Encephalitis | Cochrane, NICE, HDAS, Embase, PubMed, Medline, Cinahl | none | ④⑤⑦ |
| Houts 2021^[37]^ | 2021 | The United States | none | F | Migraine | PubMed | none | ① |
| Mateus 2021^[38]^ | 2021 | Portugal | CRD42020143429 | F | Myotonic dystrophy type 1 | Web of Science, PubMed, Embase | Quality Assessment Tool for Observational Cohort and Cross-Sectional Studies | ④⑩ |
| Oliveira 2020^[39]^ | 2020 | Brazil | http://doi.org/10.11124/JBISRIR-D-19- 00178 | F | Multiple sclerosis | PubMed, Scopus, Cochrane | none | ① |
| Venda 2020^[40]^ | 2020 | The United Kingdom | CRD42018118675 | F | Trigeminal neuralgia | Medline, Embase, Cochrane, Cinahl, PsycINFO | none | ④ |
| Mcdaid 2019^[41]^ | 2019 | The United Kingdom | CRD42016034067 | F | Non-respiratory sleep disturbances in children | ASSIA，Central，Cochrane | none | ② |
| Al Wattar 2015^[42]^ | 2015 | The United Kingdom | none | F | Epilepsy | Medline, Embase, Cinahl, AMED, Cochrane | Newcastle–Ottawa Scale/Cochrane Risk-of-Bias tool for randomized trials | ①④⑤ |
| Solebo 2019^[43]^ | 2019 | The United Kingdom | none | G | Childhood uveitis | ClinicalTrials. gov | none | ① |
| Johansson 2018^[44]^ | 2018 | Sweden | CRD42016039703 | H | Conductive and mixed hearing loss | Medline, Cochrane Library, Embase, Cinahl, PsycInfo, Science Direct, ISI | none | ①⑤⑦⑧ |
| Alexander 2021^[45]^ | 2021 | New Zealand | CRD42020170849 | [I](http://www.a-hospital.com/w/%E5%BE%AA%E7%8E%AF%E7%B3%BB%E7%BB%9F) | Abdominal aortic aneurysm | Medline, Embase | none | ①⑦ |
| Machin 2021^[46]^ | 2021 | The United Kingdom | CRD42019130119 | I | Abdominal aortic aneurysm | Medline, Embase | none | ① |
| Drury 2018^[47]^ | 2018 | The United Kingdom | CRD42017080205 | I | Heart lesions | Medline, Central, LILACS | Cochrane Risk-of-Bias tool for randomized trials | ①③ |
| Benstoem 2015^[48]^ | 2015 | Germany | none | I | Coronary heart disease | Cochrane | none | ① |
| Qiu 2019^[49]^ | 2019 | China | none | I | Non-valvular atrial fibrillation | PubMed, Cochrane, Wan Fang, CNKI, SinoMed | MOMENT study scoring system/JADAD scale/Newcastle-Ottawa Scale | ①⑧ |
| Yanez 2022^[50]^ | 2022 | The United Kingdom | none | I | Degenerative cervical myelopathy | Medline, Embase | COSMIN checklist | ① |
| Ambler 2020^[51]^ | 2020 | The United Kingdom | CRD42017059329 | I | Peripheral vascular disease | Medline, Embase | none | ①②④⑦⑨ |
| Andersen 2019^[52]^ | 2019 | Australia | COMET study ID 747 | I | Aneurysmal subarachnoid hemorrhage | Medline, Embase, Cinahl, Central | none | ① |
| Vanpeppen 2007^[53]^ | 2007 | The Netherlands | none | I | Stroke | Medline, Cinahl, Embase, Cochrane, DocOnline, PEDro | Physiotherapy Evidence Database (PEDro) criteria | ①⑥ |
| Ahmad 2019^[54]^ | 2019 | The United Kingdom | none | J | Asthma | Medline, Embase, Web of Science | none | ①② |
| Hinkelbein 2019^[55]^ | 2019 | Germany | none | J | Asthma | Medline | none | ①⑧ |
| Garcia 2015^[56]^ | 2015 | Australia | CRD42014007019 | J | Asthma | PubMed, Scopus, Web of Science, Scielo | none | ①② |
| Souto-Miranda 2022^[57]^ | 2022 | Portugal | CRD42017079935 | J | Chronic obstructive pulmonary disease | Scopus, Cochrane, EBSCO, Science Direct, PubMed | none | ①③⑧ |
| Mathioudakis 2019^[58]^ | 2019 | The United Kingdom | CRD42016052437 | J | Chronic obstructive pulmonary disease | Medline, PubMed | none | ①⑥ |
| Soni-jaiswal 2017^[59]^ | 2017 | The United Kingdom | COMET study ID 1447 | J | Chronic rhinosinusitis | Cochrane | none | ①⑥ |
| Rosala-Hallas 2022^[60]^ | 2022 | The United Kingdom | none | J | Bronchiolitis | Medline, Embase, Central | none | ① |
| Mcleod 2020^[61]^ | 2020 | Australia | CRD42020151785 | J | Cystic fibrosis | Medline, Embase, Cochrane | none | ⑧ |
| Qiu 2020^[62]^ | 2020 | China | none | J | COVID-19 | ChiCTR, ClinicalTrials.gov | none | ①② |
| Schuering 2020^[63]^ | 2020 | The Netherlands | none | J | Adductor spasmodic dysphonia | PubMed, Embase, Cochrane | COSMIN checklist | ①⑧ |
| Iliodromiti 2019^[64]^ | 2019 | The United Kingdom | none | J | Menopausal vasomotor symptoms | Medline, Embase, Cochran | JADAD scale/MOMENT study scoring system | ① |
| Machielsen 2021^[65]^ | 2021 | The Netherlands | CRD42018102778 | K | Cryptoglandular anal fistula | Medline, Embase, Cochrane | none | ①⑤⑥⑦⑧ |
| Nagelkerke 2019^[66]^ | 2019 | The Netherlands | none | K | Pediatric chronic intestinal failure | Cochrane, Embase, Medline | A criteria list for quality assessment of RCTs/Newcastle–Ottawa Scale | ①④⑤ |
| Rubin 2016^[67]^ | 2016 | Canada | CRD42013003798 | K | Pediatric eosinophilic esophagitis | Medline, Embase, Cochrane, Central, Cinahl | Cochrane Risk-of-Bias tool for randomized trials | ① |
| Longchamp 2020^[68]^ | 2020 | Switzerland | none | K | Haemorrhoidal disease | Medline, Pubmed, Embase, Cochrane | none | ①④ |
| Heemskerk 2019^[69]^ | 2019 | The Netherlands | none | K | Slow-transit constipation | Medline, Embase, PsycINFO | STROBE/TREND/CONSORT/MINORS | ①②④⑤ |
| Zeevenhooven 2019^[70]^ | 2019 | The Netherlands | none | K | Abdominal pain disorders | Cochrane, Medline, Embase, Cinahl | A criteria list for quality assessment of RCTs | ① |
| Barber 2018^[71]^ | 2018 | The United Kingdom | none | K | Periodontitis | 6 Databases | none | ① |
| Chapman 2018^[72]^ | 2018 | The United Kingdom | CRD42017082351 | K | Ileus | Medline, Embase, Cinahl, EBSCOhost, Cochrane | none | ① |
| Ma 2018^[73]^ | 2018 | Canada | none | K | Eosinophilic esophagitis | Medline, Embase, Central | none | ① |
| Mellor 2018^[74]^ | 2018 | The United Kingdom | CRD42017065538 | K | Small bowel obstruction | Medline, Embase, Cochrane | none | ①④ |
| Vantol 2018^[75]^ | 2018 | The Netherlands | none | K | Hemorrhoidal disease | Medline, Embase, Cochrane | none | ① |
| Levey 2017^[76]^ | 2017 | The United Kingdom | CRD42015025310 | K | Carious lesions | Embase, CNKI, PubMed, Central | none | ① |
| Singendonk 2017^[77]^ | 2017 | The Netherlands | none | K | Gastroesophageal reflux disease | Cochrane, Embase, Medline, PubMed | A criteria list for quality assessment of RCTs | ① |
| Kuizenga 2016^[78]^ | 2016 | The Netherlands | none | K | Functional Constipation in Children | Medline, Embase, Cochrane | A criteria list for quality assessment of RCTs | ① |
| Hall 2015^[79]^ | 2015 | The United Kingdom | none | K | Appendicitis | Medline, Embase, Cochrane | none | ①⑥ |
| Sharif 2015^[80]^ | 2015 | The United Kingdom | COMET study ID 795 | K | Traumatic dental injuries | Medline, Cochrane, Embase | none | ① |
| Fransen 2019^[81]^ | 2019 | The Netherlands | COMET study ID 1134 | L | Dermatological diseases | Medline, Embase | none | ①④⑦⑨ |
| Schmitt 2018^[82]^ | 2018 | Germany | none | L | Dermatological diseases | Cochrane | Cochrane Risk-of-Bias tool for randomized trials | ① |
| Reynolds 2020^[83]^ | 2020 | The United States | none | L | Actinic keratosis | PubMed, Embase, Cinahl, Cochrane | none | ① |
| Dovell 2021^[84]^ | 2021 | The United Kingdom | CRD42019128250 | L | Foot ulceration | Medline, Embase, Cochrane | none | ①② |
| Lu 2020^[85]^ | 2020 | Canada | COMET study ID 1436 | L | Pyoderma gangrenosum | Medline, Embase | COSMIN checklist | ① |
| Smith 2020^[86]^ | 2020 | The United Kingdom | none | L | Acne | Medline, Embase, PsychInfo | none | ① |
| Rönsch 2019^[87]^ | 2019 | Germany | none | L | Hand eczema | Medline, Embase, Cochrane | none | ① |
| Busard 2018^[88]^ | 2018 | The Netherlands | COMET study ID 1009 | L | Psoriasis | Cochrane, Central, Medline, Embase, LILACS | none | ① |
| Van den 2018^[89]^ | 2018 | Belgium | none | L | Incontinence-associated dermatitis | PubMed, Cinahl, Cochrane | none | ① |
| Lopez-Olivo 2020^[90]^ | 2020 | The United States | none | M | Rheumatoid arthritis | Medline, Embase | none | ⑧ |
| Minnock 2017^[91]^ | 2017 | Ireland | none | M | Rheumatoid arthritis | Medline, Cinahl, Cochrane, PsycINFO | Critical Appraisal Skills Program (CASP) checklist | ①④ |
| Koyuncu 2020^[92]^ | 2020 | Denmark | none | M | Hip and knee arthroplasty | Pubmed, Embase, Central | none | ① |
| El-Boghdadly 2019^[93]^ | 2019 | The United Kingdom | CRD42017070213 | M | Shoulder pain | Medline, PubMed, Embase, Cochrane Central, Web of Science, Cinahl | JADAD scale | ① |
| Page 2019^[94]^ | 2019 | Australia | CRD42017082628 | M | Shoulder disorders | Medline, Embase, Cinahl, SportDiscus, PsycINFO | Critical Appraisal Skills Programme (CASP) checklist | ⑪ |
| Page 2015^[95]^ | 2015 | Australia | COMET study ID 600 | M | Shoulder pain | Cochrane, Medline, Embase, Cinahl, ICTRP | none | ① |
| Marson 2021^[96]^ | 2021 | The United Kingdom | COMET study ID 1812 | M | Childhood limb fractures | Medline, Embase, Cochrane, ICTRP | none | ①③ |
| Marson 2020^[97]^ | 2020 | The United Kingdom | CRD420181066058 | M | Childhood fractures | Medline, Embase, Cochrane | none | ①③ |
| Grävare 2022^[98]^ | 2022 | The United States | CRD42020156763 | M | Tendinopathy | Embase, MEDLINE, Cochrane, CINAHL, SPORTDiscus | none | ①②④⑦ |
| Copeland 2021^[99]^ | 2021 | Canada | CRD42018095881 | M | Thumb carpometacarpal joint osteoarthritis | Medline, Embase, Cochrane, Cinahl | none | ①⑧ |
| See 2021^[100]^ | 2021 | Singapore | none | M | Low back pain | Medline, Pubmed, Academic Search Complete, PsycINFO, Embase, Cochrane, ClinicalKey | none | ⑩ |
| Cai 2020^[101]^ | 2020 | New Zealand | CRD42019137075 | M | Inflammatory arthritis | Medline, PubMed, Embase, Cochrane | none | ①④⑤⑩ |
| Crosby 2020^[102]^ | 2020 | The United Kingdom | none | M | Wrist fractures | Cochrane, Medline, SCOPUS | none | ①④⑤⑦ |
| Karpinski 2020^[103]^ | 2020 | Canada | COMET study ID 1515 | M | Dupuytren’s disease | Medline, Ovid Embase, Cinahl | none | ①⑦ |
| Rochau 2020^[104]^ | 2020 | Australia | none | M | Myelodysplastic syndromes | ClinicalTrials. gov. | none | ⑧ |
| Twohig 2020^[105]^ | 2020 | The United Kingdom | CRD42017080058 | M | Polymyalgia rheumatica | Medline, Cinahl, Embase, Web of Science, Cochrane, ClinicalTrials. gov, ISCTRN | Quality In Prognosis Studies (QUIPS) tool/Cochrane Risk-of-Bias tool for randomized trials | ①④⑤⑩ |
| Rodgers 2014^[106]^ | 2014 | The United Kingdom | none | M | Primary frozen shoulder | Medline, Embase | Tool developed by Hawker | ①②⑥⑦ |
| Marks 2013^[107]^ | 2013 | Switzerland | Netherlands National Trial Register(no. 2602) | M | Trapeziometacarpal osteoarthritis | PubMed, Embase, Web of Science, Cochrane, Cinahl, Academic search Premier, ScienceDirect, PEDro | none | ①⑧ |
| Howe 2012^[108]^ | 2012 | The United Kingdom | none | M | Knee pain | Medline, Embase, AMED, Cinahl | none | ① |
| Loganathan 2022^[109]^ | 2022 | The United Kingdom | none | N | Stress urinary incontinence | Cochrane, PubMed, Medline, Embase | none | ① |
| Yee cheung 2020^[110]^ | 2020 | The United Kingdom | CRD42019132512 | N | Stress urinary incontinence in women | Medline, Embase, Cochrane | none | ①③ |
| Doumouchtsis 2019^[111]^ | 2019 | The United Kingdom | CRD42017062237 | N | Stress urinary incontinence | Cochrane, Pubmed, Medline, Embase | JADAD scale | ① |
| Sautenet 2016^[112]^ | 2016 | France | none | N | Chronic kidney disease | Cochrane, PubMed, Medline | none | ① |
| Sadownik 2018^[113]^ | 2018 | Canada | CRD42016038918 | N | Vulvodynia | PubMed, PsycINFO | none | ①⑦ |
| Simpson 2013^[114]^ | 2013 | The United Kingdom | none | N | Vulval disease | Medline, Embase, Cochrane | none | ① |
| Duane 2022^[115]^ | 2022 | Ireland | CRD42017080205 | N | Urinary tract infection | Cochrane, PubMed, Embase | none | ①⑥ |
| Ghai 2020^[116]^ | 2020 | The United Kingdom | CRD42019134858 | N | Pelvic pain | Cochrane, Embase, Medline | JADAD scale/MOMENT study scoring system | ① |
| Wuytack 2019^[117]^ | 2019 | Ireland | COMET study ID 958 | N | Pelvic girdle pain and lumbopelvic pain | PubMed, Cochrane, PEDro, Embase | none | ①②⑥ |
| Moussa 2022^[118]^ | 2022 | The United Kingdom | COMET study ID 981 | N | Idiopathic overactive bladder | Medline, Embase, Cochrane, ICTRP | JADAD scale/MOMENT study scoring system | ① |
| Rimmer 2022^[119]^ | 2022 | The United Kingdom | unregistered | N | Male infertility | Cochrane, Cinahl, Embase, Medline, PsycINFO | none | ① |
| Tellum 2021^[120]^ | 2021 | Norway | CRD42020177466 | N | Adenomyosis | Medline, Embase, Cochrane | The Evidence Project risk of bias tool/MOMENT study scoring system | ①④ |
| Christmas 2020^[121]^ | 2020 | The United States | none | N | Genitourinary syndrome of menopause | PubMed, Scopus, Cochrane Central Register of Controlled Trials | JADAD scale | ① |
| Smith 2018^[122]^ | 2018 | The United Kingdom | CRD42016036349 | N | Miscarriage | Medline, Embase, Cinahl, Cochrane, ClinicalTrials. gov | Cochrane Risk-of-Bias tool for randomized trials | ① |
| Hirsch 2016^[123]^ | 2016 | The United Kingdom | none | N | Endometriosis | Cochrane, Embase, Medline | JADAD scale/MOMENT study scoring system | ① |
| Briscoe 2019^[124]^ | 2019 | The United States | none | O | Cesarean deliveries | Cochrane, Medline | none | ① |
| Kgosidialwa 2021^[125]^ | 2021 | Ireland | CRD42020173549 | O | Pregestational diabetes | Central, Web of Science, Medline, Cinahl, Embase | none | ① |
| Duffy 2017^[126]^ | 2017 | The United Kingdom | none | O | Pre-eclampsia | Cochrane, CINHAL, Medline, Embase, PsycINFO | none | ① |
| Smith 2014^[127]^ | 2014 | Ireland | none | O | Pregnant women | Cochrane | none | ① |
| Perry 2018^[128]^ | 2018 | The United Kingdom | CRD42016043999 | O | Twin–twin transfusion syndrome | Cochrane, Embase, Medline | JADAD scale/Newcastle–Ottawa Scale | ①⑧ |
| O'Reilly 2020^[129]^ | 2020 | Ireland | none | O | Diabetes after pregnancy | Medline, Embase, Cinahl, Cochrane | none | ①②③ |
| Dadouch 2019^[130]^ | 2019 | Canada | CRD42017080279 | O | Obesity in pregnancy | Medline, Embase, Central, ClinicalTrials. gov | MOMENT study scoring system | ①② |
| Kim 2021^[131]^ | 2021 | Australia | CRD42017065147 | O | Complications of pregnancy | Pubmed, Embase, Cochrane | none | ④ |
| Koot 2018^[132]^ | 2018 | The Netherlands | none | O | Hyperemesis gravidarum | Medline, Embase, Cochrane | MOMENT study scoring system | ① |
| Leow 2020^[133]^ | 2020 | The United Kingdom | CRD42019133104 | O | Singleton pregnancy | Medline, Embase, Cochrane | MOMENT study scoring system | ④ |
| Malinowski 2019^[134]^ | 2019 | Canada | CRD42016037333 | O | Perinatal iron deficiency anemia | Medline, Embase, Cochrane, Cinahl | JADAD scale | ① |
| Pergialiotis 2018^[135]^ | 2018 | Greece | CRD42017077375 | O | Childbirth trauma | Cochrane, Medline, Embase | JADAD scale | ① |
| Villani 2020^[136]^ | 2020 | Canada | CRD42018087837 | O | Patients with vasa previa | Medline, Embase, Cochrane, PubMed, ClinicalTrials. gov | none | ④⑤⑦⑨ |
| Webbe 2019^[137]^ | 2019 | The United Kingdom | CRD42016042110 | P | Neonatal | Central, Cinahl, Embase, Medline | none | ① |
| Webbe 2018^[138]^ | 2018 | The United Kingdom | CRD42016037874 | P | Neonatal | Medline, Cinahl, Embase, PsycINFO, ASSIA | none | ⑪ |
| Gadhvi 2020^[139]^ | 2020 | The United Kingdom | COMET study ID 1513 | P | Pediatric critical | Medline, Embase, Cinahl, AMED, BNI, HBE, HMIC, PsychINFO | none | ① |
| Townsend 2019^[140]^ | 2019 | The United Kingdom | CRD42018074910 | P | Fetal growth restriction | Embase, Medline, Cochrane | JADAD scale | ① |
| Leunbach 2019^[141]^ | 2019 | The United Kingdom | COMET study ID 1482 | Q | Hypospadias | Medline | none | ①④⑤⑦ |
| Ross 2016^[142]^ | 2016 | The United Kingdom | COMET study ID 746 | Q | Gastroschisis | Medline, Cochrane | none | ①⑥ |
| Tsichlaki 2014^[143]^ | 2014 | The United States | none | Q | Malocclusion | Medline, Embase, Cinahl, Cochrane | none | ① |
| Boric 2019^[144]^ | 2019 | Croatia | none | R | Postoperative pain | Medline, Cochrane, DARE, Cinahl, PsycINFO | none | ① |
| Boric 2018^[145]^ | 2018 | Croatia | CRD42015029654 | R | Postoperative pain | Medline, Cochrane Database of Systematic Reviews, DARE, Cinahl, PsycINFO | none | ①③ |
| Ross 2018^[146]^ | 2018 | The United States | none | R | Postoperative pain in paediatric and adolescent patients | ClinicalTrials. gov | none | ①② |
| Bera 2021^[147]^ | 2021 | The United Kingdom | CRD42018109487 | R | End-stage organ failure | Medline, Embase, Central, Web of Science, ClinicalTrials. gov | Cochrane Risk-of-Bias tool for randomized trials | ① |
| Henning 2021^[148]^ | 2021 | Denmark | CRD42020155565 | R | Hyperhidrosis | Cochrane, Embase, PubMed | Diagnostic Accuracy Studies-2 (QUADAS-2) | ⑧⑩ |
| Maeßen 2021^[149]^ | 2021 | Germany | CRD42018095137 | R | Acute postoperative pain | PubMed, Medline, Embase, Central | none | ① |
| Taverny 2019^[150]^ | 2019 | France | CRD42016036575 | R | Critically ill patients | PubMed, Medline, Cochrane | Cochrane Risk-of-Bias tool for randomized trials | ① |
| Whitehead 2015^[151]^ | 2015 | The United Kingdom | none | R | Cardiac arrest | Medline, Embase, Cinahl, Cochrane | none | ① |
| Khan 2022^[152]^ | 2022 | The United Kingdom | CRD42017062456 | S | Pelvic organ prolapse | Cochrane, Pubmed, Medline, Embase | MOMENT study scoring system/JADAD scale/Newcastle-Ottawa Scale | ① |
| De Mattos 2018^[153]^ | 2018 | Brazil | CRD42017062456 | S | Pelvic organ prolapse | Cochrane, Embase, Medline | JADAD scale/MOMENT study scoring system | ① |
| Abouyannis 2021^[154]^ | 2021 | Nigeria | CRD42020196160 | S | Snake bites | Medline, Cochrane, Web of Science, Embase | none | ① |
| Bayuo 2020^[155]^ | 2020 | Hong Kong (China) | none | S | Burns rehabilitation | Cochrane, Embase, Cinahl, EBSCOHost, PubMed, Web of Science, Science Direct | none | ①②③④⑤⑦⑨ |
| Beuscart 2017^[156]^ | 2017 | Belgium | none | S | Polypharmacy patients | [http://apps.who.int/trialsearch/, https://www.clinicaltrialsregister.eu/ctr-search/search, ClinicalTrials. gov](http://apps.who.int/trialsearch/,%20https:/www.clinicaltrialsregister.eu/ctr-search/search,%20ClinicalTrials.%20gov) | none | ① |
| De Mattos 2019^[157]^ | 2019 | Brazil | CRD42017062456 | S | Apical prolapse | Cochrane, Embase, Medline, Scopus | MOMENT study scoring system | ① |
| Lourenço 2020^[158]^ | 2020 | Brazil | CRD42017062456 | S | Posterior vaginal prolapse | Cochrane, Embase, PubMed, Medline, Scopus | JADAD scale | ① |
| Deshmukh 2021^[159]^ | 2021 | The United Kingdom | CRD42019126299 | S | Hand fractures and joint injuries | MEDLIN, Embase, Cochrane, Central, PubMed, Cinahl, PEDro, PsycINFO | nine point classification system | ①③ |
| Naughton 2018^[160]^ | 2018 | The United States | none | S | Hand diseases | PubMed, PEDro, OTseeker, ProQuest | Physiotherapy Evidence Database (PEDro) criteria | ① |
| Venkatesh 2022^[161]^ | 2022 | Australia | unregistered | S | Patients with burns | PubMed, Medline | none | ①④ |
| Miller 2021^[162]^ | 2021 | The United Kingdom | unregistered | S | Traumatic brachial plexus injuries | Medline, Embase, Cinahl, AMED | nine point classification system | ①②④⑦⑨ |
| Murphy,R 2021^[163]^ | 2021 | The United Kingdom | CRD42018103001 | S | Peripheral nerve injury of the upper limb | Medline, Embase, Allied and Complementary Medicine Database, Cochrane | none | ①④⑤⑦ |
| McKeown 2019^[164]^ | 2019 | The United Kingdom | none | S | Ankle fractures | Medline, Embase, Cinahl, AMED, Cochrane | none | ①③ |
| Young 2019^[165]^ | 2019 | The United Kingdom | CRD42017060908 | S | Cutaneous burn | 4 Databases | none | ① |
| Zebis 2019^[166]^ | 2019 | Denmark | none | S | Anterior cruciate ligament injuries in children | PubMed, Embase, Cinahl, PEDro | none | ①②③④⑤⑦⑧⑨ |
| Morris 2018^[167]^ | 2018 | The United Kingdom | none | S | Tibial fracture | Medline, Embase, PubMed, Cinahl, PsycINFO, Medline | COSMIN checklist | ①⑦ |
| Oner 2016^[168]^ | 2016 | The Netherlands | none | S | Spine trauma | Medline, Embase, Cochrane | none | ①⑨ |
| Post 2010^[169]^ | 2010 | The Netherlands | none | S | Spinal cord injury | Medline, Embase, PsycINFO, Cinahl | none | ① |
| Richardson 2022a^[170]^ | 2022 | Australia | CRD42019140793 | T | Recessive or X-linked condition | Medline, Embase, Cinahl, PsycINFO | QualSyst tool | ⑪ |
| Richardson 2022^[171]^ | 2022 | Australia | CRD42019140793 | T | Recessive or X-linked condition | Medline, Embase, Cinahl, PsycINFO | QualSyst tool | ⑪ |
| Walker 2021^[172]^ | 2021 | The United Kingdom | none | T | Breech birth at term | Medline, Embase, Cochrane | MOMENT study scoring (four of these questions) | ①⑥ |
| Hassan 2019^[173]^ | 2019 | Canada | CRD42018099069 | T | Open rhinoplasty | Medline, Epub Ahead of Print, InNAProcess & Other NonNAIndexed Citations | Oxford Centre for Evidence-Based Medicine Levels of Evidence | ①④⑤⑨ |
| Shorter 2019^[174]^ | 2019 | The United Kingdom | CRD42016047185 | T | Drinkers | Medline, Embase, PsycINFO, HMIC, Cinahl, AMED, Cochrane, ERIC, ClinicalTrials. gov, ICTRP | none | ① |
| Agha 2015^[175]^ | 2015 | The United Kingdom | none | T | Breast reconstruction | 20 Databases | none | ④⑦⑨ |

A: Certain infectious or parasitic diseases. B: Neoplasms. C: Diseases of the immune system. D: Endocrine, nutritional or metabolic diseases. E: Mental, behavioural or neurodevelopmental disorders. F: Diseases of the nervous system. G: Diseases of the visual system. H: Diseases of the ear or mastoid process. I: Diseases of the circulatory system. J: Diseases of the respiratory system. K: Diseases of the digestive system. L: Diseases of the skin. M: Diseases of the musculoskeletal system or connective tissue. N: Diseases of the genitourinary system. O: Pregnancy, childbirth or the puerperium. P: Certain conditions originating in the perinatal period. Q: Developmental anomalies. R: Symptoms, signs or clinical findings, not elsewhere classified. S: Injury, poisoning or certain other consequences of external causes. T: Factors influencing health status or contact with health services. ①: Randomized controlled trial. ②: Non-randomized controlled trial. ③: Quasi-randomized controlled trial. ④: Cohort study. ⑤: Case-control study. ⑥: Systematic review. ⑦: Case Series Study. ⑧:Observational study. ⑨: Case Report. ⑩: Cross-sectional study. ⑪ : Qualitative methods (e.g. focus groups, Delphi methods, nominal group techniques, participant observation, interviews). NR: Not Reported.

# Table S2: The main steps in formulating COS.

| **ID** | | **Step I Step II** | | **Step III** | **Step IV** |
| --- | --- | --- | --- | --- | --- |
|  | Reynolds 2021^[9]^ | SR | semi-structured interviews | Delphi survey | consensus meeting |
|  | Alkhaffaf 2018^[10]^ | SR | semi-structured interviews | Delphi survey | consensus meeting |
|  | Fish 2018^[11]^ | SR | semi-structured interviews | Delphi survey | consensus meeting |
|  | Rose 2021^[19]^ | SR | semi-structured interviews | Delphi survey | consensus meeting |
|  | Rose 2020^[20]^ | SR | semi-structured interviews | Delphi survey | consensus meeting |
|  | Kelly 2020^[25]^ | SR | semi-structured interviews | Delphi survey | consensus meeting |
|  | Qiu 2019^[49]^ | SR | semi-structured interviews | Delphi survey | consensus meeting |
|  | Mathioudakis 2019^[58]^ | SR | semi-structured interviews | Delphi survey | consensus meeting |
|  | Rosala-Hallas 2022^[60]^ | SR | semi-structured interviews | Delphi survey | consensus meeting |
|  | Marson 2021^[96]^ | SR | semi-structured interviews | Delphi survey | consensus meeting |
|  | Marson 2020^[97]^ | SR | semi-structured interviews | Delphi survey | consensus meeting |
|  | Wuytack 2019^[117]^ | SR | semi-structured interviews | Delphi survey | consensus meeting |
|  | Dadouch 2019^[130]^ | SR | semi-structured interviews | Delphi survey | consensus meeting |
|  | Kim 2021^[131]^ | SR | semi-structured interviews | Delphi survey | consensus meeting |
|  | Beuscart 2017^[156]^ | SR | semi-structured interviews | Delphi survey | consensus meeting |
|  | Miller 2021^[162]^ | SR | semi-structured interviews | Delphi survey | consensus meeting |
|  | Barnes 2019^[5]^ | SR | Delphi survey | - | - |
|  | Addington 2012^[27]^ | SR | Delphi survey | - | - |
|  | Al Wattar 2015^[42]^ | SR | Delphi survey | - | - |
|  | Rochau 2020^[104]^ | SR | Delphi survey | - | - |
|  | Tellum 2021^[120]^ | SR | Delphi survey | - | - |
|  | Hirsch 2016^[123]^ | SR | Delphi survey | - | - |
|  | Briscoe 2019^[124]^ | SR | Delphi survey | - | - |
|  | Duffy 2017^[126]^ | SR | Delphi survey | - | - |
|  | Webbe 2018^[138]^ | SR | Delphi survey | - | - |
|  | Hellberg 2021^[23]^ | SR | Delphi survey | consensus meeting | - |
|  | Qiu 2020^[62]^ | SR | Delphi survey | consensus meeting | - |
|  | Reynolds 2020^[83]^ | SR | Delphi survey | consensus meeting | - |
|  | Kgosidialwa 2021^[125]^ | SR | Delphi survey | consensus meeting | - |
|  | Webbe 2019^[137]^ | SR | Delphi survey | consensus meeting | - |
|  | Townsend 2019^[140]^ | SR | Delphi survey | consensus meeting | - |
|  | Matvienko-sikar 2018^[14]^ | SR | Focus groups | Delphi survey | consensus meeting |
|  | Ambler 2020^[51]^ | SR | Focus groups | Delphi survey | consensus meeting |
|  | Chapman 2018^[72]^ | SR | Focus groups | Delphi survey | consensus meeting |
|  | Murphy, R 2021^[163]^ | SR | Focus groups | Delphi survey | consensus meeting |
|  | Walker 2021^[172]^ | SR | Focus groups | Delphi survey | consensus meeting |
|  | Harman 2021^[13]^ | SR | semi-structured interviews /Focus groups | Delphi survey | consensus meeting |
|  | Morris 2014^[29]^ | SR | semi-structured interviews /Focus groups | Delphi survey | consensus meeting |
|  | Richardson 2022^[30]^ | SR | semi-structured interviews /Focus groups | Delphi survey | consensus meeting |
|  | Rönsch 2019^[87]^ | SR | semi-structured interviews /Focus groups | Delphi survey | consensus meeting |
|  | Hatemi 2014^[34]^ | SR | semi-structured interviews /Focus groups | Delphi survey | - |
|  | Webster 2017^[22]^ | SR | Focus groups | consensus meeting | - |

SR: Systematic review.

**Table S3: The quality assessment results of the included systematic reviews by AMSTAR 2.0**

| **ID** | **Item 1** | **Item 2^*^** | **Item 3** | **Item 4^*^** | **Item 5** | **Item 6** | **Item 7^*^** | **Item 8** | **Item 9^*^** | **Item 10** | **Item 11^*^** | **Item 12** | **Item 13^*^** | **Item 14** | **Item 15^*^** | **Item 16** | **Rating** |
| --- | --- | --- | --- | --- | --- | --- | --- | --- | --- | --- | --- | --- | --- | --- | --- | --- | --- |
| Henry 2022^[1]^ | Y | Y | N | N | Y | Y | PY | PY | N | N | NA | NA | N | Y | NA | Y | critically low |
| Barnes 2019^[2]^ | Y | N | N | PY | Y | Y | PY | PY | N | N | NA | NA | N | N | NA | Y | critically low |
| Lee, S 2019^[3]^ | Y | N | N | PY | Y | Y | PY | Y | Y | N | NA | NA | N | Y | NA | Y | critically low |
| Lee, A 2021^[4]^ | Y | Y | N | PY | Y | Y | PY | PY | N | N | NA | NA | N | Y | NA | Y | critically low |
| Potter 2010^[5]^ | Y | N | Y | PY | Y | Y | PY | Y | Y | N | NA | NA | Y | Y | NA | Y | low |
| Fledderus 2019^[6]^ | Y | Y | N | PY | Y | Y | PY | Y | N | N | NA | NA | N | Y | NA | Y | critically low |
| Amoah 2022^[7]^ | Y | Y | Y | PY | Y | Y | PY | PY | Y | Y | NA | NA | N | Y | NA | Y | low |
| O’Connor 2021^[8]^ | Y | N | N | PY | Y | Y | Y | Y | N | N | NA | NA | N | Y | NA | Y | critically low |
| Reynolds 2021^[9]^ | Y | N | N | PY | Y | Y | PY | PY | N | N | NA | NA | N | N | NA | Y | critically low |
| Alkhaffaf 2018^[10]^ | Y | N | Y | PY | Y | Y | PY | Y | N | Y | NA | NA | N | Y | NA | Y | critically low |
| Fish 2018^[11]^ | Y | Y | N | PY | Y | Y | PY | Y | N | N | NA | NA | N | Y | NA | Y | critically low |
| Aiyegbusi 2022^[12]^ | Y | N | N | PY | Y | Y | PY | Y | Y | Y | NA | NA | N | Y | NA | Y | critically low |
| Harman 2021^[13]^ | Y | Y | N | PY | Y | Y | PY | Y | N | N | NA | NA | N | Y | NA | Y | critically low |
| Matvienko-sikar 2018^[14]^ | Y | Y | N | PY | Y | Y | PY | Y | N | N | NA | NA | N | Y | NA | Y | critically low |
| Hopkins 2014^[15]^ | Y | N | N | PY | Y | Y | N | PY | N | N | NA | NA | N | Y | NA | Y | critically low |
| Li 2021^[16]^ | Y | Y | N | PY | Y | Y | PY | Y | N | N | NA | NA | N | Y | NA | Y | critically low |
| Harman 2017^[17]^ | Y | Y | N | N | Y | Y | PY | Y | N | N | NA | NA | N | Y | NA | Y | critically low |
| Agar 2021^[18]^ | Y | N | N | PY | Y | Y | PY | Y | Y | Y | NA | NA | Y | Y | NA | Y | low |
| Rose 2021^[19]^ | Y | N | N | N | Y | Y | PY | PY | N | N | NA | NA | N | N | NA | Y | critically low |
| Rose 2020^[20]^ | Y | N | N | PY | Y | Y | PY | PY | Y | N | NA | NA | Y | Y | NA | Y | low |
| Goncalves 2018^[21]^ | Y | Y | N | PY | Y | Y | PY | Y | Y | N | NA | NA | N | Y | NA | Y | low |
| Webster 2017^[22]^ | Y | Y | N | PY | Y | Y | PY | PY | N | N | NA | NA | N | N | NA | Y | critically low |
| Hellberg 2021^[23]^ | Y | N | Y | PY | Y | Y | PY | Y | N | N | NA | NA | N | N | NA | Y | critically low |
| Mew 2020^[24]^ | Y | N | Y | PY | Y | Y | PY | Y | N | N | NA | NA | N | Y | NA | Y | critically low |
| Kelly 2020^[25]^ | Y | N | N | N | Y | Y | PY | N | N | N | NA | NA | N | Y | NA | Y | critically low |
| Shan 2020^[26]^ | Y | Y | N | PY | Y | Y | PY | Y | N | N | NA | NA | N | Y | NA | Y | critically low |
| Addington 2012^[27]^ | Y | N | N | PY | Y | Y | N | PY | N | Y | NA | NA | N | N | NA | Y | critically low |
| Kapadia 2016^[28]^ | Y | Y | N | PY | Y | Y | PY | Y | N | N | NA | NA | N | Y | NA | Y | critically low |
| Morris 2014^[29]^ | Y | N | N | PY | Y | Y | PY | Y | N | N | NA | NA | N | Y | NA | Y | critically low |
| Cup 2007^[30]^ | Y | N | N | PY | Y | Y | PY | PY | Y | N | NA | NA | N | N | NA | Y | critically low |
| Deckert 2015^[31]^ | Y | N | N | PY | Y | Y | PY | Y | N | N | NA | NA | N | Y | NA | Y | critically low |
| Gallo 2020^[32]^ | Y | Y | N | PY | Y | Y | PY | Y | N | N | NA | NA | N | Y | NA | Y | critically low |
| Grieve 2015^[33]^ | Y | N | N | PY | Y | Y | N | Y | N | N | NA | NA | N | N | NA | Y | critically low |
| Hatemi 2014^[34]^ | Y | N | N | N | Y | Y | PY | PY | N | N | NA | NA | N | Y | NA | Y | critically low |
| Jerosch-herold 2006^[35]^ | Y | N | Y | PY | Y | Y | N | Y | N | N | NA | NA | N | N | NA | Y | critically low |
| Tooren 2022^[36]^ | Y | N | N | PY | Y | Y | PY | Y | N | N | NA | NA | N | N | NA | N | critically low |
| Houts 2021^[37]^ | Y | N | N | N | Y | Y | PY | PY | N | N | NA | NA | N | Y | NA | Y | critically low |
| Mateus 2021^[38]^ | Y | Y | N | PY | Y | Y | Y | Y | Y | N | NA | NA | N | Y | NA | Y | low |
| Oliveira 2020^[39]^ | Y | Y | N | PY | Y | Y | PY | PY | N | N | NA | NA | N | Y | NA | Y | critically low |
| Venda 2020^[40]^ | Y | Y | N | N | Y | Y | PY | PY | N | N | NA | NA | N | Y | NA | Y | critically low |
| Mcdaid 2019^[41]^ | Y | Y | N | PY | Y | Y | N | PY | N | N | NA | NA | N | Y | NA | Y | critically low |
| Al Wattar 2015^[42]^ | Y | N | N | PY | Y | Y | Y | PY | Y | Y | NA | NA | Y | Y | NA | Y | low |
| Solebo 2019^[43]^ | Y | N | N | N | Y | Y | Y | PY | N | N | NA | NA | N | Y | NA | Y | critically low |
| Johansson 2018^[44]^ | Y | Y | N | PY | Y | Y | PY | PY | N | N | NA | NA | N | N | NA | Y | critically low |
| Alexander 2021^[45]^ | Y | Y | N | PY | Y | Y | PY | PY | N | N | NA | NA | N | Y | NA | Y | critically low |
| Machin 2021^[46]^ | Y | Y | N | PY | Y | Y | PY | PY | N | N | NA | NA | N | Y | NA | N | critically low |
| Drury 2018^[47]^ | Y | Y | Y | PY | Y | Y | PY | PY | Y | N | NA | NA | Y | Y | NA | Y | high |
| Benstoem 2015^[48]^ | Y | N | Y | N | Y | Y | PY | PY | N | N | NA | NA | N | Y | NA | Y | critically low |
| Qiu 2019^[49]^ | Y | N | N | PY | Y | Y | PY | Y | Y | N | NA | NA | N | Y | NA | Y | critically low |
| Yanez 2022^[50]^ | Y | N | N | PY | Y | Y | PY | Y | N | N | NA | NA | Y | Y | NA | Y | critically low |
| Ambler 2020^[51]^ | Y | Y | N | PY | Y | Y | N | PY | N | Y | NA | NA | N | N | NA | Y | critically low |
| Andersen 2019^[52]^ | Y | Y | Y | PY | Y | Y | PY | Y | N | N | NA | NA | N | Y | NA | Y | critically low |
| Vanpeppen 2007^[53]^ | Y | N | N | PY | Y | Y | N | PY | N | N | NA | NA | N | N | NA | Y | critically low |
| Ahmad 2019^[54]^ | Y | N | N | PY | Y | Y | PY | Y | N | Y | NA | NA | N | Y | NA | Y | critically low |
| Hinkelbein 2019^[55]^ | Y | N | N | N | Y | Y | Y | PY | N | N | NA | NA | N | Y | NA | Y | critically low |
| Garcia 2015^[56]^ | Y | Y | Y | PY | Y | Y | Y | Y | N | Y | NA | NA | N | Y | NA | Y | critically low |
| Souto-Miranda 2022^[57]^ | Y | Y | N | PY | Y | Y | PY | Y | N | N | NA | NA | N | N | NA | Y | critically low |
| Mathioudakis 2019^[58]^ | Y | Y | N | PY | Y | Y | PY | Y | N | N | NA | NA | N | Y | NA | Y | critically low |
| Soni-jaiswal 2017^[59]^ | Y | Y | N | N | Y | Y | PY | Y | N | N | NA | NA | N | Y | NA | Y | critically low |
| Rosala-Hallas 2022^[60]^ | Y | N | N | PY | Y | Y | PY | Y | N | N | NA | NA | N | N | NA | Y | critically low |
| Mcleod 2020^[61]^ | Y | Y | N | PY | Y | Y | PY | Y | N | N | NA | NA | N | Y | NA | Y | critically low |
| Qiu 2020^[62]^ | Y | N | N | PY | Y | Y | N | N | N | N | NA | NA | N | N | NA | Y | critically low |
| Schuering 2020^[63]^ | Y | N | N | PY | Y | Y | PY | PY | N | N | NA | NA | N | Y | NA | Y | critically low |
| Iliodromiti 2019^[64]^ | Y | N | Y | PY | Y | Y | PY | PY | Y | N | NA | NA | N | Y | NA | Y | critically low |
| Machielsen 2021^[65]^ | Y | Y | N | PY | Y | Y | PY | Y | N | N | NA | NA | N | Y | NA | Y | critically low |
| Nagelkerke 2019^[66]^ | Y | N | N | PY | Y | Y | Y | Y | Y | N | NA | NA | N | Y | NA | Y | critically low |
| Rubin 2016^[67]^ | Y | Y | N | PY | Y | Y | PY | PY | Y | N | NA | NA | Y | Y | NA | Y | moderate |
| Longchamp 2020^[68]^ | Y | N | N | PY | Y | Y | Y | Y | N | N | NA | NA | N | Y | NA | Y | critically low |
| Heemskerk 2019^[69]^ | Y | N | N | PY | Y | Y | PY | Y | N | N | NA | NA | N | Y | NA | Y | critically low |
| Zeevenhooven 2019^[70]^ | Y | N | N | PY | Y | Y | Y | Y | Y | N | NA | NA | N | Y | NA | N | critically low |
| Barber 2018^[71]^ | Y | N | Y | Y | Y | Y | PY | PY | N | Y | NA | NA | N | Y | NA | Y | critically low |
| Chapman 2018^[72]^ | Y | Y | N | PY | Y | Y | PY | Y | N | N | NA | NA | N | Y | NA | Y | critically low |
| Ma 2018^[73]^ | Y | N | Y | PY | Y | Y | N | Y | N | N | NA | NA | Y | Y | NA | Y | critically low |
| Mellor 2018^[74]^ | Y | Y | N | PY | Y | Y | PY | Y | N | N | NA | NA | N | Y | NA | Y | critically low |
| Vantol 2018^[75]^ | Y | N | N | PY | Y | Y | PY | Y | N | N | NA | NA | N | Y | NA | Y | critically low |
| Levey 2017^[76]^ | Y | Y | N | PY | Y | Y | PY | PY | N | N | NA | NA | N | Y | NA | Y | critically low |
| Singendonk 2017^[77]^ | Y | N | N | PY | Y | Y | Y | Y | Y | N | NA | NA | N | Y | NA | Y | critically low |
| Kuizenga 2016^[78]^ | Y | N | Y | PY | Y | Y | Y | PY | Y | N | NA | NA | N | Y | NA | Y | critically low |
| Hall 2015^[79]^ | Y | N | Y | PY | Y | Y | Y | Y | N | N | NA | NA | N | Y | NA | Y | critically low |
| Sharif 2015^[80]^ | Y | Y | N | PY | Y | Y | PY | Y | N | N | NA | NA | N | Y | NA | N | critically low |
| Fransen 2019^[81]^ | Y | Y | N | PY | Y | Y | PY | Y | N | N | NA | NA | N | N | NA | Y | critically low |
| Schmitt 2018^[82]^ | Y | N | N | N | Y | Y | Y | Y | Y | N | NA | NA | Y | Y | NA | Y | critically low |
| Reynolds 2020^[83]^ | Y | N | N | PY | Y | Y | PY | PY | N | N | NA | NA | N | N | NA | Y | critically low |
| Dovell 2021^[84]^ | Y | Y | N | PY | Y | Y | PY | PY | N | N | NA | NA | N | Y | NA | Y | critically low |
| Lu 2020^[85]^ | Y | Y | N | PY | Y | Y | PY | PY | N | N | NA | NA | Y | Y | NA | Y | low |
| Smith 2020^[86]^ | Y | N | N | PY | Y | Y | PY | Y | N | N | NA | NA | N | N | NA | Y | critically low |
| Rönsch 2019^[87]^ | Y | N | N | PY | Y | Y | PY | Y | N | N | NA | NA | N | N | NA | Y | critically low |
| Busard 2018^[88]^ | Y | Y | Y | PY | Y | Y | PY | Y | N | N | NA | NA | N | Y | NA | Y | critically low |
| Van den 2018^[89]^ | Y | N | N | PY | Y | Y | N | PY | N | N | NA | NA | N | N | NA | Y | critically low |
| Lopez-Olivo 2020^[90]^ | Y | N | N | PY | Y | Y | PY | Y | N | N | NA | NA | N | Y | NA | Y | critically low |
| Minnock 2017^[91]^ | Y | N | Y | PY | Y | Y | PY | Y | Y | N | NA | NA | N | N | NA | Y | critically low |
| Koyuncu 2020^[92]^ | Y | N | N | N | Y | Y | PY | PY | N | N | NA | NA | N | Y | NA | Y | critically low |
| El-Boghdadly 2019^[93]^ | Y | Y | Y | PY | Y | Y | PY | PY | Y | N | NA | NA | Y | Y | NA | Y | high |
| Page 2019^[94]^ | Y | Y | N | N | Y | Y | N | PY | Y | N | NA | NA | N | N | NA | N | critically low |
| Page 2015^[95]^ | Y | Y | N | PY | Y | Y | PY | PY | N | N | NA | NA | N | N | NA | Y | critically low |
| Marson 2021^[96]^ | Y | Y | N | PY | Y | Y | N | N | N | N | NA | NA | N | N | NA | Y | critically low |
| Marson 2020^[97]^ | Y | Y | Y | PY | Y | Y | PY | Y | N | N | NA | NA | N | Y | NA | Y | critically low |
| Grävare 2022^[98]^ | Y | Y | N | PY | Y | Y | PY | PY | N | N | NA | NA | N | Y | NA | Y | critically low |
| Copeland 2021^[99]^ | Y | Y | Y | PY | Y | Y | PY | Y | N | Y | NA | NA | N | N | NA | Y | critically low |
| See 2021^[100]^ | Y | N | N | PY | Y | Y | PY | Y | N | N | NA | NA | N | Y | NA | Y | critically low |
| Cai 2020^[101]^ | Y | Y | N | PY | Y | Y | Y | Y | N | N | NA | NA | N | Y | NA | Y | critically low |
| Crosby 2020^[102]^ | Y | N | N | PY | Y | Y | PY | Y | N | Y | NA | NA | N | Y | NA | Y | critically low |
| Karpinski 2020^[103]^ | Y | Y | N | PY | Y | Y | N | PY | N | N | NA | NA | N | Y | NA | Y | critically low |
| Rochau 2020^[104]^ | Y | N | N | N | Y | Y | PY | Y | N | N | NA | NA | N | N | NA | Y | critically low |
| Twohig 2020^[105]^ | Y | Y | N | PY | Y | Y | PY | Y | Y | N | NA | NA | Y | Y | NA | Y | moderate |
| Rodgers 2014^[106]^ | Y | N | N | PY | Y | Y | N | Y | N | N | NA | NA | N | Y | NA | N | critically low |
| Marks 2013^[107]^ | Y | Y | N | PY | Y | Y | PY | Y | N | N | NA | NA | Y | N | NA | Y | low |
| Howe 2012^[108]^ | Y | N | N | PY | Y | Y | PY | Y | N | N | NA | NA | N | N | NA | Y | critically low |
| Loganathan 2022^[109]^ | Y | N | Y | PY | Y | Y | PY | PY | N | N | NA | NA | N | Y | NA | Y | critically low |
| Yee cheung 2020^[110]^ | Y | Y | Y | PY | Y | Y | PY | Y | N | N | NA | NA | N | Y | NA | Y | critically low |
| Doumouchtsis 2019^[111]^ | Y | Y | Y | PY | Y | Y | PY | PY | Y | N | NA | NA | N | Y | NA | Y | low |
| Sautenet 2016^[112]^ | Y | N | N | PY | Y | Y | PY | Y | N | N | NA | NA | N | Y | NA | Y | critically low |
| Sadownik 2018^[113]^ | Y | Y | N | PY | Y | Y | PY | PY | N | N | NA | NA | N | Y | NA | Y | critically low |
| Simpson 2013^[114]^ | Y | N | Y | PY | Y | Y | PY | PY | N | N | NA | NA | N | Y | NA | Y | critically low |
| Duane 2022^[115]^ | Y | Y | N | PY | Y | Y | Y | Y | N | N | NA | NA | N | Y | NA | Y | critically low |
| Ghai 2020^[116]^ | Y | Y | Y | PY | Y | Y | PY | Y | Y | N | NA | NA | N | Y | NA | Y | low |
| Wuytack 2019^[117]^ | Y | Y | N | PY | Y | Y | Y | Y | N | N | NA | NA | N | Y | NA | Y | critically low |
| Moussa 2022^[118]^ | Y | Y | Y | PY | Y | Y | N | PY | Y | Y | NA | NA | N | Y | NA | Y | critically low |
| Rimmer 2022^[119]^ | Y | PY | Y | PY | Y | Y | PY | PY | N | N | NA | NA | N | Y | NA | Y | critically low |
| Tellum 2021^[120]^ | Y | Y | Y | PY | Y | Y | PY | Y | Y | N | NA | NA | Y | Y | NA | Y | high |
| Christmas 2020^[121]^ | Y | N | N | PY | Y | Y | PY | Y | Y | N | NA | NA | Y | Y | NA | Y | low |
| Smith 2018^[122]^ | Y | Y | Y | PY | Y | Y | PY | Y | Y | N | NA | NA | Y | Y | NA | Y | high |
| Hirsch 2016^[123]^ | Y | N | Y | PY | Y | Y | PY | Y | Y | Y | NA | NA | N | Y | NA | Y | critically low |
| Briscoe 2019^[124]^ | Y | N | N | PY | Y | Y | Y | PY | N | N | NA | NA | N | N | NA | Y | critically low |
| Kgosidialwa 2021^[125]^ | Y | Y | N | PY | Y | Y | PY | PY | N | N | NA | NA | N | Y | NA | Y | critically low |
| Duffy 2017^[126]^ | Y | N | N | PY | Y | Y | PY | PY | N | N | NA | NA | N | N | NA | Y | critically low |
| Smith 2014^[127]^ | Y | N | N | N | Y | Y | PY | PY | N | N | NA | NA | N | N | NA | N | critically low |
| Perry 2018^[128]^ | Y | Y | Y | PY | Y | Y | Y | PY | Y | Y | NA | NA | N | Y | NA | N | low |
| O'Reilly 2020^[129]^ | Y | N | N | PY | Y | Y | Y | Y | N | N | NA | NA | N | Y | NA | Y | critically low |
| Dadouch 2019^[130]^ | Y | Y | Y | PY | Y | Y | PY | Y | N | N | NA | NA | N | Y | NA | Y | critically low |
| Kim 2021^[131]^ | Y | Y | N | PY | Y | Y | PY | Y | N | N | NA | NA | N | Y | NA | Y | critically low |
| Koot 2018^[132]^ | Y | N | N | PY | Y | Y | PY | Y | N | N | NA | NA | N | Y | NA | Y | critically low |
| Leow 2020^[133]^ | Y | Y | Y | PY | Y | Y | PY | Y | N | N | NA | NA | N | Y | NA | Y | critically low |
| Malinowski 2019^[134]^ | Y | Y | Y | PY | Y | Y | PY | Y | Y | N | NA | NA | N | Y | NA | Y | low |
| Pergialiotis 2018^[135]^ | Y | Y | N | PY | Y | Y | PY | PY | Y | Y | NA | NA | N | Y | NA | Y | low |
| Villani 2020^[136]^ | Y | Y | N | PY | Y | Y | PY | Y | N | N | NA | NA | N | Y | NA | Y | critically low |
| Webbe 2019^[137]^ | Y | Y | N | PY | Y | Y | PY | PY | N | N | NA | NA | N | Y | NA | Y | critically low |
| Webbe 2018^[138]^ | Y | Y | N | PY | Y | Y | PY | Y | N | N | NA | NA | N | N | NA | Y | critically low |
| Gadhvi 2020^[139]^ | Y | Y | Y | PY | Y | Y | PY | PY | N | N | NA | NA | N | Y | NA | Y | critically low |
| Townsend 2019^[140]^ | Y | Y | N | PY | Y | Y | Y | Y | Y | Y | NA | NA | N | Y | NA | N | low |
| Leunbach 2019^[141]^ | Y | Y | N | N | Y | Y | N | PY | N | N | NA | NA | N | N | NA | Y | critically low |
| Ross 2016^[142]^ | Y | Y | Y | PY | Y | Y | PY | Y | N | N | NA | NA | N | Y | NA | Y | critically low |
| Tsichlaki 2014^[143]^ | Y | N | N | PY | Y | Y | PY | Y | N | N | NA | NA | N | N | NA | Y | critically low |
| Boric 2019^[144]^ | Y | N | Y | PY | Y | Y | N | Y | N | Y | NA | NA | N | Y | NA | Y | critically low |
| Boric 2018^[145]^ | Y | Y | N | N | Y | Y | PY | PY | N | Y | NA | NA | N | N | NA | Y | critically low |
| Ross 2018^[146]^ | Y | N | N | N | Y | Y | Y | PY | N | N | NA | NA | N | Y | NA | Y | critically low |
| Bera 2021^[147]^ | Y | Y | N | PY | Y | Y | PY | Y | Y | Y | NA | NA | Y | Y | NA | Y | high |
| Henning 2021^[148]^ | Y | Y | N | PY | Y | Y | PY | PY | Y | N | NA | NA | Y | Y | NA | Y | moderate |
| Maeßen 2021^[149]^ | Y | Y | Y | PY | Y | Y | Y | PY | N | N | NA | NA | N | Y | NA | Y | critically low |
| Taverny 2019^[150]^ | Y | Y | N | PY | Y | Y | PY | Y | Y | Y | NA | NA | Y | Y | NA | Y | high |
| Whitehead 2015^[151]^ | Y | N | Y | PY | Y | Y | PY | Y | N | N | NA | NA | N | Y | NA | Y | critically low |
| Khan 2022^[152]^ | Y | Y | Y | PY | Y | Y | Y | Y | Y | N | NA | NA | N | Y | NA | Y | low |
| De Mattos 2018^[153]^ | Y | Y | Y | PY | Y | Y | PY | PY | Y | N | NA | NA | N | Y | NA | Y | low |
| Abouyannis 2021^[154]^ | Y | Y | N | PY | Y | Y | PY | Y | N | Y | NA | NA | N | Y | NA | Y | critically low |
| Bayuo 2020^[155]^ | Y | N | N | Y | Y | Y | PY | Y | N | Y | NA | NA | N | N | NA | Y | critically low |
| Beuscart 2017^[156]^ | Y | N | N | PY | Y | Y | PY | Y | N | N | NA | NA | N | Y | NA | Y | critically low |
| De Mattos 2019^[157]^ | Y | Y | N | PY | Y | Y | PY | Y | N | N | NA | NA | N | Y | NA | Y | critically low |
| Lourenço 2020^[158]^ | Y | Y | Y | PY | Y | Y | PY | Y | Y | Y | NA | NA | N | Y | NA | Y | low |
| Deshmukh 2021^[159]^ | Y | Y | N | PY | Y | Y | PY | Y | N | N | NA | NA | Y | Y | NA | Y | low |
| Naughton 2018^[160]^ | Y | N | N | PY | Y | Y | PY | Y | N | N | NA | NA | N | N | NA | Y | critically low |
| Venkatesh 2022^[161]^ | Y | PY | Y | PY | Y | Y | PY | Y | N | N | NA | NA | N | N | NA | Y | critically low |
| Miller 2021^[162]^ | Y | PY | N | PY | Y | Y | PY | Y | N | N | NA | NA | Y | Y | NA | Y | low |
| Murphy,R 2021^[163]^ | Y | Y | Y | PY | Y | Y | PY | Y | N | N | NA | NA | N | Y | NA | Y | critically low |
| McKeown 2019^[164]^ | Y | N | Y | PY | Y | Y | PY | Y | N | N | NA | NA | N | Y | NA | Y | critically low |
| Young 2019^[165]^ | Y | Y | N | PY | Y | Y | PY | Y | N | N | NA | NA | N | Y | NA | Y | critically low |
| Zebis 2019^[166]^ | Y | N | N | PY | Y | Y | PY | PY | N | N | NA | NA | N | N | NA | Y | critically low |
| Morris 2018^[167]^ | Y | N | N | PY | Y | Y | PY | PY | N | N | NA | NA | N | N | NA | Y | critically low |
| Oner 2016^[168]^ | Y | N | N | PY | Y | Y | PY | PY | N | N | NA | NA | N | Y | NA | Y | critically low |
| Post 2010^[169]^ | Y | N | Y | PY | Y | Y | N | PY | N | N | NA | NA | N | Y | NA | Y | critically low |
| Richardson 2022a^[170]^ | Y | Y | N | PY | Y | Y | PY | Y | Y | N | NA | NA | Y | Y | NA | Y | moderate |
| Richardson 2022^[171]^ | Y | Y | N | PY | Y | Y | PY | Y | Y | N | NA | NA | Y | Y | NA | Y | moderate |
| Walker 2021^[172]^ | Y | N | Y | PY | Y | Y | PY | Y | N | N | NA | NA | N | Y | NA | Y | critically low |
| Hassan 2019^[173]^ | Y | Y | N | PY | Y | Y | PY | PY | Y | N | NA | NA | N | Y | NA | Y | low |
| Shorter 2019^[174]^ | Y | Y | Y | PY | Y | Y | PY | Y | N | N | NA | NA | N | Y | NA | Y | critically low |
| Agha 2015^[175]^ | Y | N | Y | N | Y | Y | N | PY | N | Y | NA | NA | N | Y | NA | Y | critically low |

Item 1: Did the research questions and inclusion criteria for the review include the components of PICO? Item 2: Did the report of the review contain an explicit statement that the review methods were established prior to the conduct of the review and did the report justify any significant deviations from the protocol? Item 3: Did the review authors explain their selection of the study designs for inclusion in the review? Item 4: Did the review authors use a comprehensive literature search strategy? Item 5: Did the review authors perform study selection in duplicate? Item 6: Did the review authors perform data extraction in duplicate? Item 7: Did the review authors provide a list of excluded studies and justify the exclusions? Item 8: Did the review authors describe the included studies in adequate detail? Item 9: Did the review authors use a satisfactory technique for assessing the risk bias in individual studies that were included in the review? Item 10: Did the review authors report on the sources of funding for the studies included in the review? Item 11: If meta-analysis was performed, did the review authors use appropriate methods for statistical combination of results? Item 12: If meta-analysis was performed, did the review authors assess the potential impact of the risk bias in individual studies on the results of meta-analysis or other evidence synthesis? Item 13: Did the review authors account for the risk bias in primary studies when interpreting/discussing the results of the review? Item 14: Did the review authors provide a satisfactory explanation for, and discussion of, any heterogeneity observed in the results of review? Item 15: If they performed quantitative syntheses did the review authors carry out an adequate investigation of publication bias (small study bias) and discuss its likely impact on the results of the review? Item 16: Did the review authors report any potential sources of conflict of interest, including any funding they received for conducting the review?

*: critical domains; Y: Yes; PY: Partial Yes; N: No; *NA*: not applicable.

**References**

[1] Henry CJ, Semova G, Barnes E, *et al.* Neonatal sepsis: a systematic review of core outcomes from randomised clinical trials. *Pediatr Res*. 2022; 91(4):735-742. https://doi.or-g/10.1038/s41390-021-01883-y.

[2] Barnes J, Hunter J, Harris S, *et al*. Systematic review and consensus definitions for the Standardised Endpoints in Perioperative Medicine (StEP) initiative: infection and sepsis. *Br J Anaesth*. 2019; 122(4):500-508. <https://doi.org/10.1016/j.bja.2019.01.009.>

[3] Lee S, Lee Y, Lee S, *et al*. Toward Developing a Standardized Core Set of Outcome Measures in Mobile Health Interventions for Tuberculosis Management: Systematic Review. *JMIR Mhealth Uhealth*. 2019;7(2):e12385. https://doi.org/10.2196/12385.

[4] Lee A, Kwasnicki RM, Khan H, *et al*. Outcome reporting in therapeutic mammaplasty: a systematic review. *BJS Open*. 2021;5(6):zrab126. <https://doi.org/10.1093/bjsope-n/zrab126.>

[5] Potter S, Brigic A, Whiting PF, *et al.* Reporting clinical outcomes of breast reconstruction: a systematic review. *J Natl Cancer Inst*. 2011;103(1):31-46. https://doi.org/10.1093/jnci/djq438.

[6] Fledderus AC, Franke CJJ, Eggen CAM, *et al*. Outcomes and measurement instruments used in congenital melanocytic naevi research: A systematic review. *J Plast Reconstr Aesthet Surg.* 2020;73(4):703-715. <https://doi.org/10.1016/j.bjps.2019.11.023.>

[7] Amoah A, Chiu S, Quinn SD. Choice of primary and secondary outcomes in randomised controlled trials evaluating treatment for uterine fibroids: a systematic review. *BJOG.* 2022;129(3):345-355. <https://doi.org/10.1111/1471-0528.16933.>

[8] O'Connor L, Smyth E, Bennett AE, *et al.* Identifying outcomes reported in exercise interventions in oesophagogastric cancer survivors: a systematic review. *BMC Cancer*. 2021;21(1):586. <https://doi.org/10.1186/s12885-021-08290-w.>

[9] Reynolds KA, Schlessinger DI, Yanes AF, *et al.* Development of a core outcome set for cutaneous squamous cell carcinoma trials: identification of core domains and outcomes. *Br J Dermatol*. 2021;184(6):1113-1122. <https://doi.org/10.1111/bjd.19693.>

[10] Alkhaffaf B, Blazeby JM, Williamson PR, *et al*. Reporting of outcomes in gastric cancer surgery trials: a systematic review. *BMJ Open*. 2018;8(10):e021796. https://doi.or-g/10.1136/bmjopen-2018-021796.

[11] Fish R, Sanders C, Ryan N, *et al*. Systematic review of outcome measures following chemoradiotherapy for the treatment of anal cancer (CORMAC). *Colorectal Dis*. 2018;20(5):371-382. https://doi.org/10.1111/codi.14103.

[12] Aiyegbusi OL, di Ruffano LF, Retzer A, *et al*. Outcome selection for tissue-agnostic drug trials for immune-mediated inflammatory diseases: a systematic review of core outcome sets and regulatory guidance. *Trials*. 2022;23(1):42. https://doi.org/10.1186/s13063-022-06000-w.

[13] Harman NL, Gorst SL, Williamson PR, *et al.* Scout - sarcoidosis outcomes taskforce. A systematic review of outcomes to inform the development of a core outcome set for pulmonary sarcoidosis. *Sarcoidosis Vasc Diffuse Lung Di*s. 2021;38(3):e2021034. https://doi.org/10.36141/svdld.v38i3.10737.

[14] Matvienko-Sikar K, Griffin C, McGrath N, *et al*. Developing a core outcome set for childhood obesity prevention: A systematic review. *Matern Child Nutr.* 2019;15(1):e12680. https://doi.org/10.1111/mcn.12680.

[15] Hopkins JC, Howes N, Chalmers K, *et al*. Outcome reporting in bariatric surgery: an in-depth analysis to inform the development of a core outcome set, the BARIACT Study. *Obes Rev*. 2015;16(1):88-106. https://doi.org/10.1111/obr.12240.

[16] Li G, Han R, Cao W, *et al*. Outcome Reporting Variability in Trials of Chinese Medicine for Hyperlipidemia: A Systematic Review for Developing a Core Outcome Set. Evid *Based Complement Alternat Med*. 2021;2021:8822215. https://doi.org/10.1155/2021/8822215.

[17] Harman NL, James R, Wilding J, *et al*. SCORE-IT study team. SCORE-IT (Selecting Core Outcomes for Randomised Effectiveness trials In Type 2 diabetes): a systematic review of registered trials. *Trials*. 2017;18(1):597. https://doi.org/10.1186/s13063-017-2317-5.

[18] Agar MR, Siddiqi N, Hosie A, *et al*. Outcomes and measures of delirium interventional studies in palliative care to inform a core outcome set: A systematic review. *Palliat Med*. 2021;35(10):1761-1775. https://doi.org/10.1177/02692163211040186.

[19] Rose L, Burry L, Agar M, *et al*. A core outcome set for studies evaluating interventions to prevent and/or treat delirium for adults requiring an acute care hospital admission: an international key stakeholder informed consensus study. *BMC Med.* 2021;19(1):143. https://doi.org/10.1186/s12916-021-02015-3.

[20] Rose L, Agar M, Burry L, *et al*. Reporting of Outcomes and Outcome Measures in Studies of Interventions to Prevent and/or Treat Delirium in the Critically Ill: A Systematic Review. *Crit Care Med*. 2020;48(4):e316-e324. https://doi.org/10.1097/CCM.0000000000004238.

[21] Gonçalves AC, Cruz J, Marques A, *et al*. Evaluating physical activity in dementia: a systematic review of outcomes to inform the development of a core outcome set. *Age Ageing*. 2018;47(1):34-41. https://doi.org/10.1093/ageing/afx135.

[22] Webster L, Groskreutz D, Grinbergs-Saull A, *et al*. Core outcome measures for interventions to prevent or slow the progress of dementia for people living with mild to moderate dementia: Systematic review and consensus recommendations. *PLoS One*. 2017;12(6):e0179521. https://doi.org/10.1093/ageing/afx135.

[23] Hellberg C, Österberg M, Jonsson AK, *et al*. Important research outcomes for treatment studies of perinatal depression: systematic overview and development of a core outcome set. *BJOG*. 2021;128(13):2141-2149. https://doi.org/10.1111/1471-0528.16780.

[24] Mew EJ, Monsour A, Saeed L, *et al*. Systematic scoping review identifies heterogeneity in outcomes measured in adolescent depression clinical trials. *J Clin Epidemiol*. 2020;126:71-79. https://doi.org/10.1016/j.jclinepi.2020.06.013.

[25] Kelly LE, Shan F, MacVicar S, *et al*. A Core Outcome Set for Neonatal Opioid Withdrawal Syndrome. *Pediatrics*. 2020;146(1):e20200018. https://doi.org/10.1542/peds.20-20-0018.

[26] Shan F, MacVicar S, Allegaert K, *et al*. Outcome reporting in neonates experiencing withdrawal following opioid exposure in pregnancy: a systematic review. *Trials*. 2020;21(1):262. https://doi.org/10.1186/s13063-020-4183-9.

[27] Addington DE, Mckenzie E, Wang J, *et al*. Development of a core set of performance measures for evaluating schizophrenia treatment services. *Psychiatr Serv*. 2012;63(6):5-84-591. https://doi.org/10.1176/appi.ps.201100453.

[28] Kapadia MZ, Joachim KC, Balasingham C, *et al*. A Core Outcome Set for Children With Feeding Tubes and Neurologic Impairment: A Systematic Review. *Pediatrics.* 2016;138(1):e20153967. https://doi.org/10.1542/peds.2015-3967.

[29] Morris C, Janssens A, Allard A, *et al*. Informing the NHS Outcomes Framework: evaluating meaningful health outcomes for children with neurodisability using multiple methods including systematic review, qualitative research, Delphi survey and consensus meeting. Southampton (UK): NIHR Journals Library; May 2014.

[30] Cup EH, Pieterse AJ, Ten Broek-Pastoor JM, *et al*. Exercise therapy and other types of physical therapy for patients with neuromuscular diseases: a systematic review. *Arch Phys Med Rehabil*. 2007;88(11):1452-1464. https://doi.org/10.1016/j.apmr.2007.07.024.

[31] Deckert S, Kaiser U, Kopkow C, *et al*. A systematic review of the outcomes reported in multimodal pain therapy for chronic pain. *Eur J Pain*. 2016;20(1):51-63. https://do-i.org/10.1002/ejp.721.

[32] Gallo L, Gallo M, Murphy J, *et al*. Reporting Outcomes and Outcome Measures in Cubital Tunnel Syndrome: A Systematic Review. *J Hand Surg Am*. 2020;45(8):707-728.e9. https://doi.org/10.1016/j.jhsa.2020.04.001.

[33] Grieve S, Jones L, Walsh N, *et al*. What outcome measures are commonly used for Complex Regional Pain Syndrome clinical trials? A systematic review of the literature. *Eur J Pain*. 2016;20(3):331-340. https://doi.org/10.1002/ejp.733.

[34] Hatemi G, Merkel PA, Hamuryudan V, *et al*. Outcome measures used in clinical trials for Behçet syndrome: a systematic review. *J Rheumatol*. 2014;41(3):599-612. https://doi.org/10.3899/jrheum.131249.

[35] Jerosch-Herold C, Leite JC, Song F. A systematic review of outcomes assessed in randomized controlled trials of surgical interventions for carpal tunnel syndrome using the International Classification of Functioning, Disability and Health (ICF) as a reference tool. *BMC Musculoskelet Disord*. 2006;7:96. https://doi.org/10.1186/1471-2474-7-96.

[36] Tooren HVD, Easton A, Hooper C, *et al*. How should we define a 'good' outcome from encephalitis? A systematic review of the range of outcome measures used in the long-term follow-up of patients with encephalitis. *Clin Med (Lond)*. 2022;22(2):145-148. https://doi.org/10.7861/clinmed.2021-0505.

[37] Houts CR, McGinley JS, Nishida TK, *et al*. Systematic review of outcomes and endpoints in acute migraine clinical trials. *Headache*. 2021;61(2):263-275. https://doi.org/1-0.1111/head.14067.

[38] Mateus T, Costa A, Viegas D, *et al*. Outcome measures frequently used to assess muscle strength in patients with myotonic dystrophy type 1: a systematic review. *Neuromuscul Disord*. 2022;32(2):99-115. https://doi.org/10.1016/j.nmd.2021.09.014.

[39] Oliveira ML, Lucchetta RC, Bonetti AF, *et al*. Efficacy outcomes reported in trials of multiple sclerosis: A systematic scoping review. *Mult Scler Relat Disord*. 2020;45:1-02435. https://doi.org/10.1016/j.msard.2020.102435.

[40] Nova CV, Zakrzewska JM, Baker SR, *et al*. Treatment Outcomes in Trigeminal Neuralgia-A Systematic Review of Domains, Dimensions and Measures. *World Neurosurg X*. 2020;6:100070. https://doi.org/10.1016/j.wnsx.2020.100070.

[41] McDaid C, Parker A, Scantlebury A, *et al.* Outcome domains and outcome measures used in studies assessing the effectiveness of interventions to manage non-respiratory sleep disturbances in children with neurodisabilities: a systematic review. *BMJ Open*. 2019;9(6):e027205. https://doi.org/10.1136/bmjopen-2018-027205.

[42] Al Wattar BH, Placzek A, Troko J, *et al.* Variation in the reporting of outcomes among pregnant women with epilepsy: a systematic review. *Eur J Obstet Gynecol Reprod Biol.* 2015;195:193-199. https://doi.org/10.1016/j.ejogrb.2015.10.017.

[43] Solebo AL, Barry RJ, Keane PA, *et al*. Under-utilisation of reproducible, child appropriate or patient reported outcome measures in childhood uveitis interventional research. *Orphanet J Rare Dis*. 2019;14(1):125. https://doi.org/10.1186/s13023-019-1108-3.

[44] Johansson ML, Tysome JR, Hill-Feltham P, *et al*. Physical outcome measures for conductive and mixed hearing loss treatment: A systematic review. *Clin Otolaryngol*. 2018;43(5):1226-1234. https://doi.org/10.1111/coa.13131.

[45] Alexander HC, Nguyen CH, Bartlett AS, *et al*. Reporting of Clinical Outcomes After Endovascular Aortic Aneurysm Repair: A Systematic Review. *Ann Vasc Surg*. 2021;77:306-314. https://doi.org/10.1016/j.avsg.2021.06.006.

[46] Machin M, Ulug P, Pandirajan K, *et al*. Towards a Core Outcome Set for Abdominal Aortic Aneurysm: Systematic Review of Outcomes Reported Following Intact and Ruptured Abdominal Aortic Aneurysm Repair. *Eur J Vasc Endovasc Surg*. 2021;61(6):909-918. https://doi.org/10.1016/j.ejvs.2021.02.009.

[47] Drury NE, Yim I, Patel AJ, *et al*. Cardioplegia in paediatric cardiac surgery: a systematic review of randomized controlled trials. *Interact Cardiovasc Thorac Surg*. 2019;28(1):144-150. https://doi.org/10.1093/icvts/ivy199.

[48] Benstoem C, Moza A, Autschbach R, *et al*. Evaluating outcomes used in cardiothoracic surgery interventional research: a systematic review of reviews to develop a core outcome set. *PLoS One*. 2015;10(4):e0122204. https://doi.org/10.1371/journal.pone.0122204.

[49] Qiu R, Hu J, Huang Y, *et al*. Outcome reporting from clinical trials of non-valvular atrial fibrillation treated with traditional Chinese medicine or Western medicine: a systematic review. *BMJ Open*. 2019;9(8):e028803. https://doi.org/10.1136/bmjopen-2018-028803.

[50] Yanez Touzet A, Bhatti A, Dohle E, *et al*. Clinical outcome measures and their evidence base in degenerative cervical myelopathy: a systematic review to inform a core measurement set (AO Spine RECODE-DCM). *BMJ Open*. 2022;12(1):e057650. https://doi.org/10.1136/bmjopen-2021-057650.

[51] Ambler GK, Brookes-Howell L, Jones JAR, *et al*. Development of Core Outcome Sets for People Undergoing Major Lower Limb Amputation for Complications of Peripheral Vascular Disease. *Eur J Vasc Endovasc Surg*. 2020;60(5):730-738. https://doi.org/10.1016/j.ejvs.2020.06.021.

[52] Andersen CR, Fitzgerald E, Delaney A, *et al*. A Systematic Review of Outcome Measures Employed in Aneurysmal Subarachnoid Hemorrhage (aSAH) Clinical Research. *Neurocrit Care*. 2019;30(3):534-541. https://doi.org/10.1007/s12028-018-0566-0.

[53] van Peppen RP, Hendriks HJ, van Meeteren NL, *et al*. The development of a clinical practice stroke guideline for physiotherapists in The Netherlands: a systematic review of available evidence. *Disabil Rehabil*. 2007;29(10):767-783. https://doi.org/10.1080/09638280600919764.

[54] Ahmad I, Onwochei DN, Muldoon S, *et al*. Airway management research: a systematic review. *Anaesthesia*. 2019;74(2):225-236. https://doi.org/10.1-111/anae.14471.

[55] Hinkelbein J, Iovino I, De Robertis E, *et al*. Outcomes in video laryngoscopy studies from 2007 to 2017: systematic review and analysis of primary and secondary endpoints for a core set of outcomes in video laryngoscopy research. *BMC Anesthesiol.* 2019;19(1):47. https://doi.org/10.1186/s12871-019-0716-8.

[56] Garcia-Cardenas V, Armour C, Benrimoj SI, *et al*. Pharmacists' interventions on clinical asthma outcomes: a systematic review. *Eur Respir J.* 2016;47(4):1134-1143. https://doi.org/10.1183/13993003.01497-2015.

[57] Souto-Miranda S, Rodrigues G, Spruit MA, *et al*. Pulmonary rehabilitation outcomes in individuals with chronic obstructive pulmonary disease: A systematic review. *Ann Phys Rehabil Med*. 2022;65(3):101564. <https://doi.org/10.1016/j.rehab.2021.101564.>

[58] Mathioudakis AG, Moberg M, Janner J, *et al*. Outcomes reported on the management of COPD exacerbations: a systematic survey of randomised controlled trials. *ERJ Open Res*. 2019;5(2):00072-2019. https://doi.org/10.1183/23120541.00072-2019.

[59] Soni-Jaiswal A, Lakhani R, Hopkins C. Developing a core outcome set for chronic rhinosinusitis: a systematic review of outcomes utilised in the current literature. *Trials*. 2017;18(1):320. https://doi.org/10.1186/s13063-017-2060-y.

[60] Rosala-Hallas A, Jones AP, Williamson PR, *et al*. Which outcomes should be used in future bronchiolitis trials? Developing a bronchiolitis core outcome set using a systematic review, Delphi survey and a consensus workshop. *BMJ Open*. 2022;12(3):e052943. https://doi.org/10.1136/bmjopen-2021-052943.

[61] McLeod C, Wood J, Schultz A, *et al.* Outcomes and endpoints reported in studies of pulmonary exacerbations in people with cystic fibrosis: A systematic review. *J Cyst Fibros.* 2020;19(6):858-867. https://doi.org/10.1016/j.jcf.2020.08.015.

[62] Qiu R, Zhao C, Liang T, *et al*. Core Outcome Set for Clinical Trials of COVID-19 Based on Traditional Chinese and Western Medicine. *Front Pharmacol*. 2020;11:781. https://doi.org/10.3389/fphar.2020.00781.

[63] Schuering JHC, van Hof KS, Heijnen BJ, *et al*. Proposal for a Core Outcome Set of Measurement Instruments to Assess Quality of Voice in Adductor Spasmodic Dysphonia Based on a Literature Review. *J Voice*. 2021;35(6):933.e7-933.e21. https://doi.org/10.1016/j.jvoice.2020.02.010.

[64] Iliodromiti S, Wang W, Lumsden MA, *et al*. Variation in menopausal vasomotor symptoms outcomes in clinical trials: a systematic review. *BJOG.* 2020;127(3):320-333. https://doi.org/10.1111/1471-0528.15990.

[65] Machielsen AJHM, Iqbal N, Kimman ML, *et al*. Heterogeneity in outcome selection, definition and measurement in studies assessing the treatment of cryptoglandular anal fistula: findings from a systematic review. *Tech Coloproctol*. 2021;25(7):761-830. https://doi.org/10.1007/s10151-021-02452-5.

[66] Nagelkerke SCJ, Mager DJ, Benninga MA, *et al*. Reporting on outcome measures in pediatric chronic intestinal failure: A systematic review. *Clin Nutr*. 2020;39(7):1992-2000. https://doi.org/10.1016/j.clnu.2019.08.027.

[67] Rubin T, Clayton J, Adams D, *et al*. Systematic review of outcome measures in pediatric eosinophilic esophagitis treatment trials. *Allergy Asthma Clin Immunol*. 2016;12(1):45. https://doi.org/10.1186/s13223-016-0144-y.

[68] Longchamp G, Liot É, Meyer J, *et al*. Scoring systems as outcomes assessment of the treatments for haemorrhoidal disease: a systematic review of the literature. *Int J Colorectal Dis*. 2020;35(6):1015-1024. https://doi.org/10.1007/s00384-020-03603-0.

[69] Heemskerk SCM, Rotteveel AH, Melenhorst J, *et al*. Heterogeneous outcome reporting in adult slow-transit constipation studies: Systematic review towards a core outcome set. *J Gastroenterol Hepatol*. 2020;35(2):192-203. https://doi.org/10.1111/jgh.14818.

[70] Zeevenhooven J, Timp ML, Singendonk MMJ, *et al*. Definitions of Pediatric Functional Abdominal Pain Disorders and Outcome Measures: A Systematic Review. *J Pediatr*. 2019;212:52-59.e16. https://doi.org/10.1016/j.jpeds.2019.04.048.

[71] Barber S, Bekker HL, Meads D, *et al*. Identification and appraisal of outcome measures used to evaluate hypodontia care: A systematic review. *Am J Orthod Dentofacial Orthop*. 2018;153(2):184-194.e18.

[72] Chapman SJ, Thorpe G, Vallance AE, *et al*. Systematic review of definitions and outcome measures for return of bowel function after gastrointestinal surgery. *BJS Open*. 2018;3(1):1-10. https://doi.org/10.1002/bjs5.102.

[73] Ma C, van Rhijn BD, Jairath V, *et al*. Heterogeneity in Clinical, Endoscopic, and Histologic Outcome Measures and Placebo Response Rates in Clinical Trials of Eosinophilic Esophagitis: A Systematic Review. *Clin Gastroenterol Hepatol*. 2018;16(11):1714-1729.e3. https://doi.org/10.1016/j.cgh.2018.06.005.

[74] Mellor K, Hind D, Lee MJ. A systematic review of outcomes reported in small bowel obstruction research. *J Surg Res.* 2018;229:41-50. https://doi.org/10.1016/j.jss.2018.0-3.044.

[75] van Tol RR, van Zwietering E, Kleijnen J, *et al*. Towards a core outcome set for hemorrhoidal disease-a systematic review of outcomes reported in literature. *Int J Colorectal Dis.* 2018;33(7):849-856. https://doi.org/10.1007/s00384-018-3046-2.

[76] Levey C, Innes N, Schwendicke F, *et al*. Outcomes in randomised controlled trials in prevention and management of carious lesions: a systematic review. *Trials*. 2017;18(1):515. https://doi.org/10.1186/s13063-017-2256-1

[77] Singendonk MMJ, Brink AJ, Steutel NF, *et al*. Variations in Definitions and Outcome Measures in Gastroesophageal Reflux Disease: A Systematic Review. *Pediatrics*. 2017;140(2):e20164166. https://doi.org/10.1542/peds.2016-4166

[78] Kuizenga-Wessel S, Heckert SL, Tros W, *et al*. Reporting on Outcome Measures of Functional Constipation in Children-A Systematic Review. *J Pediatr Gastroenterol Nutr.* 2016;62(6):840-846. https://doi.org/10.1097/MPG.0000000000001110

[79] Hall NJ, Kapadia MZ, Eaton S, *et al.* Outcome reporting in randomised controlled trials and meta-analyses of appendicitis treatments in children: a systematic review. *Trials*. 2015;16:275. https://doi.org/10.1186/s13063-015-0783-1

[80] Sharif MO, Tejani-Sharif A, Kenny K, *et al*. A systematic review of outcome measures used in clinical trials of treatment interventions following traumatic dental injuries. *Dent Traumatol*. 2015;31(6):422-428. https://doi.org/10.1111/edt.12227

[81] Fransen F, Tio DCKS, Prinsen CAC, *et al*. A systematic review of outcome reporting in laser treatments for dermatological diseases. *J Eur Acad Dermatol Venereol*. 2020;34(1):47-53. https://doi.org/10.1111/jdv.15928

[82] Schmitt J, Lange T, Kottner J, *et al*. Cochrane Reviews and Dermatological Trials Outcome Concordance: Why Core Outcome Sets Could Make Trial Results More Usable. *J Invest Dermatol.* 2019;139(5):1045-1053. https://doi.org/10.1016/j.jid.2018.11.019

[83] Reynolds KA, Schlessinger DI, Vasic J, *et al.* Core Outcome Set for Actinic Keratosis Clinical Trials. *JAMA Dermatol*. 2020;156(3):326-333. https://doi.org/10.1001/jamadermatol.2019.4212

[84] Dovell G, Staniszewska A, Ramirez J, *et al*. A systematic review of outcome reporting for interventions to treat people with diabetic foot ulceration. *Diabet Med*. 2021;38(10):e14664. https://doi.org/10.1111/dme.14664

[85] Lu JD, Hobbs MM, Huang WW, *et al*. Identification and evaluation of outcome measurement instruments in pyoderma gangrenosum: a systematic review. *Br J Dermatol*. 2020;183(5):821-828. https://doi.org/10.1111/bjd.19027

[86] Smith H, Layton AM, Thiboutot D, *et al*. Identifying the Impacts of Acne and the Use of Questionnaires to Detect These Impacts: A Systematic Literature Review. *Am J Clin Dermatol*. 2021;22(2):159-171. https://doi.org/10.1007/s40257-020-00564-6

[87] Rönsch H, Apfelbacher C, Brans R, *et al*. Which outcomes have been measured in hand eczema trials? A systematic review. *Contact Dermatitis*. 2019;80(4):201-207. https://doi.org/10.1111/cod.13212

[88] Busard CI, Nolte JYC, Pasch MC, *et al*. Reporting of outcomes in randomized controlled trials on nail psoriasis: a systematic review. *Br J Dermatol*. 2018;178(3):640-649. https://doi.org/10.1111/bjd.15831

[89] Van den Bussche K, Kottner J, Beele H, *et al*. Core outcome domains in incontinence-associated dermatitis research. *J Adv Nurs.* 2018;74(7):1605-1617. https://doi.org/10.1111/jan.13562

[90] Lopez-Olivo MA, Zogala RJ, des Bordes J, *et al.* Outcomes Reported in Prospective Long-Term Observational Studies and Registries of Patients With Rheumatoid Arthritis Worldwide: An Outcome Measures in Rheumatology Systematic Review. *Arthritis Care Res (Hoboken)*. 2021;73(5):649-657. https://doi.org/10.1002/acr.24163

[91] Minnock P, McKee G, Kelly A, *et al.* Nursing sensitive outcomes in patients with rheumatoid arthritis: A systematic literature review. *Int J Nurs Stud*. 2018;77:115-129. https://doi.org/10.1016/j.ijnurstu.2017.09.005

[92] Koyuncu S, Friis CP, Laigaard J, *et al*. A systematic review of pain outcomes reported by randomised trials of hip and knee arthroplasty. *Anaesthesia*. 2021;76(2):261-269. https://doi.org/10.1111/anae.15118

[93] El-Boghdadly K, Abdallah FW, Short A, Vorobeichik L, *et al*. Outcome Selection and Methodological Quality of Major and Minor Shoulder Surgery Studies: A Scoping Review. *Clin Orthop Relat Res*. 2019;477(3):606-619. https://doi.org/10.1097/CORR.0000000000000578

[94] Page MJ, O'Connor DA, Malek M, et al. Patients' experience of shoulder disorders: a systematic review of qualitative studies for the OMERACT Shoulder Core Domain Set. *Rheumatology (Oxford)*. 2019; kez046. https://doi.org/10.1093/rheumatology/kez046

[95] Page MJ, McKenzie JE, Green SE, *et al*. Core domain and outcome measurement sets for shoulder pain trials are needed: systematic review of physical therapy trials. *J Clin Epidemiol*. 2015;68(11):1270-1281. https://doi.org/10.1016/j.jclinepi.2015.06.006

[96] Marson BA, Manning JC, James M, *et al*. Development of the CORE-Kids core set of outcome domains for studies of childhood limb fractures. *Bone Joint J*. 2021;103-B(12):1821-1830. https://doi.org/10.1302/0301-620X.103B.BJJ-2020-2321.R2

[97] Marson BA, Craxford S, Deshmukh SR, *et al*. Outcomes reported in trials of childhood fractures: a systematic review. *Bone Jt Open*. 2020;1(5):167-174. https://doi.org/1-0.1302/2633-1462.15.BJO-2020-0031

[98] Grävare Silbernagel K, Malliaras P, de Vos RJ, *et al*. ICON 2020-International Scientific Tendinopathy Symposium Consensus: A Systematic Review of Outcome Measures Reported in Clinical Trials of Achilles Tendinopathy. *Sports Med.* 2022;52(3):613-641. https://doi.org/10.1007/s40279-021-01588-6

[99] Copeland A, Gallo L, Weber C, *et al*. Reporting Outcomes and Outcome Measures in Thumb Carpometacarpal Joint Osteoarthritis: A Systematic Review. *J Hand Surg Am.* 2021;46(1):65.e1-65.e11. https://doi.org/10.1016/j.jhsa.2020.05.024

[100] See YKC, Smith HE, Car LT, *et al*. Health literacy and health outcomes in patients with low back pain: a scoping review. *BMC Med Inform Decis Mak.* 2021;21(1):215. https://doi.org/10.1186/s12911-021-01572-0

[101] Cai K, Fuller A, Hensey O, *et al*. Outcome domains reported in calcium pyrophosphate deposition studies: A scoping review by the OMERACT CPPD working group. *Semin Arthritis Rheum.* 2020;50(4):719-727. https://doi.org/10.1016/j.semarthrit.2020.05.015

[102] Crosby BT, Behbahani A, Olujohungbe O, *et al*. Developing a core outcome set for paediatric wrist fractures: a systematic review of prior outcomes. *Bone Jt Open*. 2020;1(5):121-130. https://doi.org/10.1302/2633-1462.15.BJO-2020-0007.R1

[103] Karpinski M, Moltaji S, Baxter C, *et al*. A systematic review identifying outcomes and outcome measures in Dupuytren's disease research. *J Hand Surg Eur Vol*. 2020;45(5):513-520. https://doi.org/10.1177/1753193420903624

[104] Rochau U, Stojkov I, Conrads-Frank A, *et al*. Development of a core outcome set for myelodysplastic syndromes - a Delphi study from the EUMDS Registry Group. *Br J Haematol*. 2020;191(3):405-417. https://doi.org/10.1111/bjh.16654

[105] Twohig H, Owen C, Muller S, *et al.* Outcomes Measured in Polymyalgia Rheumatica and Measurement Properties of Instruments Considered for the OMERACT Core Outcome Set: A Systematic Review. *J Rheumatol.* 2021;48(6):883-893. https://doi.org/10.3899/jrheum.200248

[106] Rodgers S, Brealey S, Jefferson L, *et al.* Exploring the outcomes in studies of primary frozen shoulder: is there a need for a core outcome set?. *Qual Life Res*. 2014;23(9):2495-2504. https://doi.org/10.1007/s11136-014-0708-6

[107] Marks M, Schoones JW, Kolling C, *et al*. Outcome measures and their measurement properties for trapeziometacarpal osteoarthritis: a systematic literature review. *J Hand Surg Eur Vol.* 2013;38(8):822-838. https://doi.org/10.1177/1753193413488301

[108] Howe TE, Dawson LJ, Syme G, *et al*. Evaluation of outcome measures for use in clinical practice for adults with musculoskeletal conditions of the knee: a systematic review. *Man Ther*. 2012;17(2):100-118. https://doi.org/10.1016/j.math.2011.07.002

[109] Loganathan J, Coffey J, Doumouchtsis SK; CHORUS: An International Collaboration for Harmonising Outcomes, Research and Standards in Urogynaecology and Women’s Health. Which patient reported outcomes (PROs) and patient reported outcome measures (PROMs) do researchers select in stress urinary incontinence surgical trials? - a systematic review. *Int Urogynecol J*. 2022;33(11):2941-2949. https://doi.org/10.1007/s00192-022-05123-7

[110] Yee Cheung F, Farag F, MacLennan S, *et al*. Is There Outcome Reporting Heterogeneity in Trials That Aim to Assess the Effectiveness of Surgical Treatments for Stress Urinary Incontinence in Women?. *Eur Urol Focus.* 2021;7(4):857-868. https://doi.org/10.1016/j.euf.2020.03.008

[111] Doumouchtsis SK, Pookarnjanamorakot P, Durnea C, *et al.* A systematic review on outcome reporting in randomised controlled trials on surgical interventions for female stress urinary incontinence: a call to develop a core outcome set. *BJOG*. 2019;126(12):1417-1422. https://doi.org/10.1111/1471-0528.15891

[112] Sautenet B, Contentin L, Bigot A, *et al*. Strong heterogeneity of outcome reporting in systematic reviews. *J Clin Epidemiol*. 2016;75:93-99. https://doi.org/10.1016/j.jclinepi.2016.01.008

[113] Sadownik LA, Yong PJ, Smith KB. Systematic Review of Treatment Outcome Measures for Vulvodynia. *J Low Genit Tract Dis*. 2018;22(3):251-259. https://doi.org/10.1097/LGT.0000000000000406

[114] Simpson RC, Thomas KS, Murphy R. Outcome measures for vulval skin conditions: a systematic review of randomized controlled trials. *Br J Dermatol*. 2013;169(3):494-501. https://doi.org/10.1111/bjd.12391

[115] Duane S, Beecher C, Vellinga A, *et al.* A systematic review of the outcomes reported in the treatment of uncomplicated urinary tract infection clinical trials. *JAC Antimicrob Resist*. 2022;4(2):dlac025. https://doi.org/10.1093/jacamr/dlac025

[116] Ghai V, Subramanian V, Jan H, *et al*. A systematic review on reported outcomes and outcome measures in female idiopathic chronic pelvic pain for the development of a core outcome set. *BJOG*. 2021;128(4):628-634. https://doi.org/10.1111/1471-0528.16412

[117] Wuytack F, O'Donovan M. Outcomes and outcomes measurements used in intervention studies of pelvic girdle pain and lumbopelvic pain: a systematic review. *Chiropr Man Therap*. 2019;27:62. https://doi.org/10.1186/s12998-019-0279-2

[118] Moussa R, Rada MP, Durnea C, *et al*. Outcome reporting in randomized controlled trials (RCTs) on the pharmacological management of idiopathic overactive bladder (OAB) in women; a systematic review for the development of core outcome sets (COS). *Int Urogynecol J*. 2022;33(5):1243-1250. https://doi.org/10.1007/s00192-021-05040-1

[119] Rimmer MP, Howie RA, Subramanian V, *et al.* Outcome reporting across randomized controlled trials evaluating potential treatments for male infertility: a systematic review. *Hum Reprod Open*. 2022;2022(2):hoac010. https://doi.org/10.1093/hropen/hoac010

[120] Tellum T, Omtvedt M, Naftalin J, *et al*. A systematic review of outcome reporting and outcome measures in studies investigating uterine-sparing treatment for adenomyosis. *Hum Reprod Open*. 2021;2021(3):hoab030. https://doi.org/10.1093/hropen/hoab030

[121] Christmas MM, Song B, Bell RJ, *et al.* Variation in outcome reporting and measurement tools in clinical trials of treatments for genitourinary symptoms in peri- and postmenopausal women: a systematic review. *Menopause*. 2020;27(9):1070-1080. https://doi.org/10.1097/GME.0000000000001570

[122] Smith PP, Dhillon-Smith RK, O'Toole E, *et al.* Outcomes in prevention and management of miscarriage trials: a systematic review. *BJOG*. 2019;126(2):176-189. https://doi.org/10.1111/1471-0528.15528

[123] Hirsch M, Duffy JMN, Kusznir JO, *et al*. Variation in outcome reporting in endometriosis trials: a systematic review. *Am J Obstet Gynecol*. 2016;214(4):452-464. https://doi.org/10.1016/j.ajog.2015.12.039

[124] Briscoe KE, Haas DM. Developing a Core Outcome Set for Cesarean Delivery Maternal Infectious Morbidity Outcomes. *Am J Perinatol*. 2020;37(4):436-452. https://doi.org/10.1055/s-0039-1681095

[125] Kgosidialwa O, Bogdanet D, Egan A, *et al.* A systematic review on outcome reporting in randomised controlled trials assessing treatment interventions in pregnant women with pregestational diabetes. *BJOG.* 2021;128(12):1894-1904. https://doi.org/10.1111/1471-0528.16842

[126] Duffy JMN, Hirsch M, Gale C, *et al*. A systematic review of primary outcomes and outcome measure reporting in randomized trials evaluating treatments for pre-eclampsia. *Int J Gynaecol Obste*t. 2017;139(3):262-267. https://doi.org/10.1002/ijgo.12298

[127] Smith V, Daly D, Lundgren I, *et al*. Salutogenically focused outcomes in systematic reviews of intrapartum interventions: a systematic review of systematic reviews. *Midwifery*. 2014;30(4):e151-e156. https://doi.org/10.1016/j.midw.2013.11.002

[128] Perry H, Duffy JMN, Umadia O, *et al.* International Collaboration to Harmonise Outcomes for Twin-Twin Transfusion Syndrome (CHOOSE). Outcome reporting across randomized trials and observational studies evaluating treatments for twin-twin transfusion syndrome: systematic review. *Ultrasound Obstet Gynecol.* 2018;52(5):577-585. https://doi.org/10.1002/uog.19068

[129] O'Reilly SL, Leonard Y, Dasgupta K, *et al*. Diabetes after pregnancy prevention trials: Systematic review for core outcome set development. *Matern Child Nutr*. 2020;16(3):e12947. https://doi.org/10.1111/mcn.12947

[130] Dadouch R, Faheim M, Susini O, *et al*. Variation in outcome reporting in studies on obesity in pregnancy-A systematic review. *Clin Obes.* 2019;9(6):e12341. https://doi.org/10.1111/cob.12341

[131] Kim BV, Aromataris EC, de Lint W, *et al*. Developing a core outcome set in interventions to prevent stillbirth: A systematic review on variations of outcome reporting. *Eur J Obstet Gynecol Reprod Biol*. 2021;259:196-206. https://doi.org/10.1016/j.ejogrb.2020.12.036

[132] Koot MH, Boelig RC, Van't Hooft J, *et al.* Variation in hyperemesis gravidarum definition and outcome reporting in randomised clinical trials: a systematic review. *BJOG.* 2018;125(12):1514-1521. https://doi.org/10.1111/1471-0528.15272

[133] Leow HW, Tan EL, Black M. Reported outcomes for planned caesarean section versus planned vaginal delivery: A systematic review. *Eur J Obstet Gynecol Reprod Biol.* 2021;256:101-108. https://doi.org/10.1016/j.ejogrb.2020.10.057

[134] Malinowski AK, D'Souza R, Khan KS, *et al*. Reported Outcomes in Perinatal Iron Deficiency Anemia Trials: A Systematic Review. *Gynecol Obstet Invest*. 2019;84(5):417-434. https://doi.org/10.1159/000495566

[135] Pergialiotis V, Durnea C, Elfituri A, *et al.* International Collaboration for Harmonising Outcomes, Research, and Standards in Urogynaecology and Women's Health (CHORUS). Do we need a core outcome set for childbirth perineal trauma research? A systematic review of outcome reporting in randomised trials evaluating the management of childbirth trauma. *BJOG*. 2018;125(12):1522-1531. https://doi.org/10.1111/1471-0528.15408

[136] Villani LA, Pavalagantharajah S, D'Souza R. Variations in reported outcomes in studies on vasa previa: a systematic review. *Am J Obstet Gynecol MFM.* 2020;2(3):100116. https://doi.org/10.1016/j.ajogmf.2020.100116

[137] Webbe JWH, Ali S, Sakonidou S, *et al.* Inconsistent outcome reporting in large neonatal trials: a systematic review. *Arch Dis Child Fetal Neonatal Ed.* 2020;105(1):69-75. https://doi.org/10.1136/archdischild-2019-316823

[138] Webbe J, Brunton G, Ali S, *et al*. Parent, patient and clinician perceptions of outcomes during and following neonatal care: a systematic review of qualitative research. *BMJ Paediatr Open*. 2018;2(1):e000343. https://doi.org/10.1136/bmjpo-2018-000343

[139] Gadhvi KR, Valla FV, Tume LN. Review of Outcomes Used in Nutrition Trials in Pediatric Critical Care. *JPEN J Parenter Enteral Nutr*. 2020;44(7):1210-1219. https://doi.org/10.1002/jpen.1765

[140] Townsend R, Sileo F, Stocker L, *et al.* Variation in outcome reporting in randomized controlled trials of interventions for prevention and treatment of fetal growth restriction. *Ultrasound Obstet Gynecol.* 2019;53(5):598-608. https://doi.org/10.1002/uog.20189

[141] Leunbach TL, O''Toole S, Springer A, *et al.* A Systematic Review of Core Outcomes for Hypospadias Surgery. *Sex Dev*. 2019;13(4):165-170. https://doi.org/10.1159/000504973

[142] Ross AR, Hall NJ. Outcome reporting in randomized controlled trials and systematic reviews of gastroschisis treatment: a systematic review. *J Pediatr Surg*. 2016;51(8):1385-1389. https://doi.org/10.1016/j.jpedsurg.2016.05.008

[143] Tsichlaki A, O'Brien K. Do orthodontic research outcomes reflect patient values? A systematic review of randomized controlled trials involving children. *Am J Orthod Dentofacial Orthop*. 2014;146(3):279-285. https://doi.org/10.1016/j.ajodo.2014.05.022

[144] Boric K, Jelicic Kadic A, Boric M, *et al.* Outcome domains and pain outcome measures in randomized controlled trials of interventions for postoperative pain in children and adolescents. *Eur J Pain.* 2019;23(2):389-396. https://doi.org/10.1002/ejp.1313

[145] Boric K, Dosenovic S, Jelicic Kadic A, *et al*. Efficacy and Safety Outcomes in Systematic Reviews of Interventions for Postoperative Pain in Children: Comparison Against the Recommended Core Outcome Set. *Pain Med*. 2018;19(11):2316-2321. https://doi.org/10.1093/pm/pnx255

[146]Ross A, Young J, Hedin R, *et al.* A systematic review of outcomes in postoperative pain studies in paediatric and adolescent patients: towards development of a core outcome set. *Anaesthesia.* 2018;73(3):375-383. https://doi.org/10.1111/anae.14211

[147] Bera KD, Shah A, English MR, *et al*. Outcome measures in solid organ donor management research: a systematic review. *Br J Anaesth.* 2021;127(5):745-759. https://doi.org/10.1016/j.bja.2021.07.008

[148] Henning MAS, Thorlacius L, Ibler KS, *et al*. How to diagnose and measure primary hyperhidrosis: a systematic review of the literature. *Clin Auton Res*. 2021;31(4):511-528. https://doi.org/10.1007/s10286-021-00794-6

[149] Maeßen TV, Austenfeld E, Kaiser U, *et al.* Systematic Review on Pain-Related Outcome Domains After Sternotomy: A First Step Toward the Development of a Core Outcome Set. *Value Health.* 2021;24(8):1203-1212. https://doi.org/10.1016/j.jval.2021.01.016

[150] Taverny G, Lescot T, Pardo E, *et al*. Outcomes used in randomised controlled trials of nutrition in the critically ill: a systematic review. *Crit Care.* 2019;23(1):12. https://doi.org/10.1186/s13054-018-2303-7

[151] Whitehead L, Perkins GD, Clarey A, *et al*. A systematic review of the outcomes reported in cardiac arrest clinical trials: the need for a core outcome set. *Resuscitation*. 2015;88:150-157. https://doi.org/10.1016/j.resuscitation.2014.11.013

[152] Khan K, Rada M, Elfituri A, *et al.* Outcome reporting in trials on conservative interventions for pelvic organ prolapse: A systematic review for the development of a core outcome set. *Eur J Obstet Gynecol Reprod Biol*. 2022;268:100-109. https://doi.org/10.1016/j.ejogrb.2021.08.028

[153] de Mattos Lourenco TR, Pergialiotis V, Duffy JMN, *et al.* A systematic review on reporting outcomes and outcome measures in trials on synthetic mesh procedures for pelvic organ prolapse: Urgent action is needed to improve quality of research. *Neurourol Urodyn*. 2019;38(2):509-524. https://doi.org/10.1002/nau.23871

[154] Abouyannis M, Aggarwal D, Lalloo DG, *et al.* Clinical outcomes and outcome measurement tools reported in randomised controlled trials of treatment for snakebite envenoming: A systematic review. *PLoS Negl Trop Dis*. 2021;15(8):e0009589. https://doi.org/10.1371/journal.pntd.0009589

[155] Bayuo J, Wong FKY. Intervention Content and Outcomes of Postdischarge Rehabilitation Programs for Adults Surviving Major Burns: A Systematic Scoping Review. *J Burn Care Res.* 2021;42(4):651-710. https://doi.org/10.1093/jbcr/iraa110

[156] Beuscart JB, Pont LG, Thevelin S, *et al.* A systematic review of the outcomes reported in trials of medication review in older patients: the need for a core outcome set. *Br J Clin Pharmacol.* 2017;83(5):942-952. https://doi.org/10.1111/bcp.13197

[157] de Mattos Lourenço TR, Pergialiotis V, Durnea C, *et al*. A systematic review of reported outcomes and outcome measures in randomized controlled trials on apical prolapse surgery. *Int J Gynaecol Obstet*. 2019;145(1):4-11. https://doi.org/10.1002/ijgo.12766

[158] Lourenço TRM, Pergialiotis V, Durnea CM, *et al.* A systematic review of reported outcomes and outcome measures in randomized trials evaluating surgical interventions for posterior vaginal prolapse to aid development of a core outcome set. *Int J Gynaecol Obstet.* 2020;148(3):271-281. https://doi.org/10.1002/ijgo.13079

[159] Deshmukh SR, Mousoulis C, Marson BA, *et al.* Core Outcome Set for Hand Fractures and Joint Injuries in Adults Group*. Developing a core outcome set for hand fractures and joint injuries in adults: a systematic review. *J Hand Surg Eur Vol*. 2021;1753193420983719. https://doi.org/10.1177/1753193420983719

[160] Naughton N, Algar L. Linking commonly used hand therapy outcome measures to individual areas of the International Classification of Functioning: A systematic review. *J Hand Ther*. 2019;32(2):243-261. https://doi.org/10.1016/j.jht.2017.11.039

[161] Venkatesh K, Henschke A, Lee RP, *et al*. Patient-centred outcomes are under-reported in the critical care burns literature: a systematic review. *Trials*. 2022;23(1):199. https://doi.org/10.1186/s13063-022-06104-3

[162] Miller C, Cross J, O'Sullivan J, *et al*. Developing a core outcome set for traumatic brachial plexus injuries: a systematic review of outcomes. *BMJ Open*. 2021;11(7):e044797. https://doi.org/10.1136/bmjopen-2020-044797

[163] Murphy RNA, Elsayed H, Singh S, *et al*. A Quantitative Systematic Review of Clinical Outcome Measure Use in Peripheral Nerve Injury of the Upper Limb. *Neurosurgery*. 2021;89(1):22-30. https://doi.org/10.1093/neuros/nyab060

[164] McKeown R, Rabiu AR, Ellard DR, *et al*. Primary outcome measures used in interventional trials for ankle fractures: a systematic review. *BMC* Musculoskelet Disord. 2019;20(1):388. https://doi.org/10.1186/s12891-019-2770-2

[165] Young AE, Davies A, Bland S, *et al*. Systematic review of clinical outcome reporting in randomised controlled trials of burn care. *BMJ Open*. 2019;9(2):e025135. https://doi.org/10.1136/bmjopen-2018-025135

[166] Zebis MK, Warming S, Pedersen MB, *et al*. Outcome Measures After ACL Injury in Pediatric Patients: A Scoping Review. *Orthop J Sports Med*. 2019;7(7):2325967119861803. https://doi.org/10.1177/2325967119861803

[167] Morris R, Pallister I, Trickett RW. Measuring outcomes following tibial fracture. *Injury*. 2019;50(2):521-533. https://doi.org/10.1016/j.injury.2018.11.025

[168] Oner FC, Jacobs WC, Lehr AM, *et al.* Toward the Development of a Universal Outcome Instrument for Spine Trauma: A Systematic Review and Content Comparison of Outcome Measures Used in Spine Trauma Research Using the ICF as Reference. *Spine (Phila Pa 1976)*. 2016;41(4):358-367. https://doi.org/10.1097/BRS.0000000000001207

[169] Post MW, Kirchberger I, Scheuringer M, *et al*. Outcome parameters in spinal cord injury research: a systematic review using the International Classification of Functioning, Disability and Health (ICF) as a reference. *Spinal Cord*. 2010;48(7):522-528. https://doi.org/10.1038/sc.2009.177

[170] Richardson E, McEwen A, Newton-John T, *et al*. Incorporating patient perspectives in the development of a core outcome set for reproductive genetic carrier screening: a sequential systematic review. *Eur J Hum Genet*. 2022;30(7):756-765. https://doi.org/10.1038/s41431-022-01090-1

[171] Richardson E, McEwen A, Newton-John T, *et al*. Systematic review of outcomes in studies of reproductive genetic carrier screening: Towards development of a core outcome set. *Genet Med.* 2022;24(1):1-14. https://doi.org/10.1016/j.gim.2021.08.005

[172] Walker S, Dasgupta T, Halliday A, *et al*. Development of a core outcome set for effectiveness studies of breech birth at term (Breech-COS): A systematic review on variations in outcome reporting. *Eur J Obstet Gynecol Reprod Biol*. 2021;263:117-126. https://doi.org/10.1016/j.ejogrb.2021.06.021

[173] Hassan Y, Leveille CF, Gallo L, *et al.* Reporting Outcomes and Outcome Measures in Open Rhinoplasty: A Systematic Review. *Aesthet Surg J*. 2020;40(2):135-146. https://doi.org/10.1093/asj/sjz138

[174] Shorter GW, Bray JW, Giles EL, *et al.* The Variability of Outcomes Used in Efficacy and Effectiveness Trials of Alcohol Brief Interventions: A Systematic Review. *J Stud Alcohol Drugs*. 2019;80(3):286-298.

[175] Agha RA, Fowler AJ, Pidgeon TE, *et al*. The Need for Core Outcome Reporting in Autologous Fat Grafting for Breast Reconstruction. *Ann Plast Surg*. 2016;77(5):506-512. https://doi.org/10.1097/SAP.0000000000000645
